# Predicting Pulsed-Laser Deposition SrTiO_3_ Homoepitaxy Growth Dynamics Using High-Speed Reflection High-Energy Electron Diffraction

**DOI:** 10.1021/acsami.4c12655

**Published:** 2025-04-08

**Authors:** Yichen Guo, Peter Meisenheimer, Shuyu Qin, Xinqiao Zhang, Julian Goddy, Ramamoorthy Ramesh, Lane W. Martin, Joshua Agar

**Affiliations:** † Department of Materials Science and Engineering, Lehigh University, Bethlehem, Pennsylvania 18015, United States; ‡ Department of Mechanical Engineering and Mechanics, Drexel University, Philadelphia, Pennsylvania 19104, United States; § Department of Materials Science and Engineering, University of California, Berkeley, California 94720, United States; ∥ Department of Computer Science and Engineering, Lehigh University, Bethlehem, Pennsylvania 18015, United States; ⊥ Materials Sciences Division, Lawrence Berkeley National Laboratory, Berkeley, California 94720, United States; # Department of Materials Science and Nanoengineering, Rice University, Houston, Texas 77005, United States; ∇ Rice Advanced Materials Institute, Rice University, Houston, Texas 77005, United States; ○ Department of Physics and Astronomy, Rice University, Houston, Texas 77005, United States

**Keywords:** pulsed-laser deposition (PLD), reflection high-energy electron diffraction (RHEED), surface reconstruction kinetics, high-speed imaging (>500 Hz), surface termination effects (TiO_2_, SrO), open-source analysis tools, machine learning and autonomous control

## Abstract

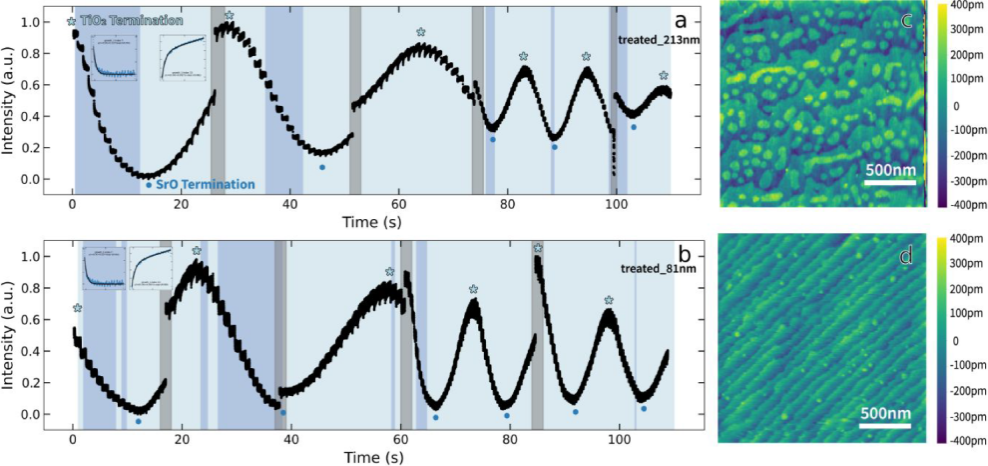

Pulsed-laser deposition (PLD) is a powerful technique for growing complex oxides with controlled stoichiometry. To understand growth dynamics therein, it is common to leverage *in situ* spectroscopies, such as reflection high-energy electron diffraction (RHEED), to monitor surface crystallinity. Most commercial systems rely on video-rate cameras operating at 60–120 Hz that lack sufficient temporal resolution to capture growth dynamics at practical deposition frequencies. Here, a high-speed platform to record *in situ* dynamics via RHEED at >500 Hz is implemented. An open-source analysis package is designed to fit diffraction spots to 2D Gaussians, allowing single-pulse surface reconstruction kinetics extraction. Using homoepitaxially deposited (001)-oriented SrTiO_3_ as a model system, we demonstrate how high-speed RHEED can provide real-time insight into growth processes obscured by slower acquisition systems. By fitting the single-pulse intensity to a set of exponential functions, we observe changes in the characteristic decay time and mechanism correlated to the substrate step width and surface termination. We observe distinct surface effects, with diffraction intensity decaying on lower-energy TiO_2_-terminated surfaces and stabilizing on SrO- or mixed-terminated surfaces. Similarly, using an exponential model, the extracted characteristic time of adatom deposition decreases with increased density of bonding sites associated with mixed termination and narrower step widths. Ultimately, this work shows how increasing RHEED temporal resolution can uncover new insights into growth processes, with practical implications for the design and control of PLD processes. This experimental platform provides new capabilities to enable data-driven machine learning analysis and autonomous control systems to enhance the complexity and fecundity of PLD.

## Introduction

Pulsed-laser deposition (PLD) is a powerful technique to grow complex oxides with controlled stoichiometry.^1−4^ In PLD, a pulsed laser ablates a target in a low-pressure (1–500 mTorr), typically oxidizing environment. The resulting plasma plume transports material, typically less than a unit cell per laser pulse, from a polycrystalline or single-crystal precursor to the substrate surface. Adatoms then diffuse along the substrate surface until they attach to the surface, producing a new nucleus or joining an existing one. Controlling material chemistries and diffusion kinetics in this way allows for the exploration of vast design spaces to optimize structure–property relationships in, typically, functional complex oxides.

Numerous studies have explored how growth processes control structure–property relationships.^5,6^ For example, in multiferroic BiFeO_3_, growth-induced defects can have a marked effect on thin-film structure and properties.^7^ Controlled ion bombardment with similar kinetic energies to those in PLD has also been used to introduce defects to alter electric properties, such as decreased electric leakage current.^7^ In PbTiO_3_, electric and dielectric properties are also highly correlated to kinetic-bombardment-induced defects during PLD.^8−10^ In a final example, ion bombardment of 0.68Pb(Mg_1/3_Nb_2/3_)O_3_-0.32PbTiO_3_ thin film results in strong interaction between point defects, weakening the relaxor behavior, thus improving dielectric properties and energy density.^11^

To date, control of synthesis processes has been chiefly empirical, requiring large changes in growth processes to obtain statistically significant and interpretable results. It is common to incorporate *in situ* diagnostics to glean further insight into growth processes, where the most common technique, reflection high-energy electron diffraction (RHEED), uses a grazing-incident electron beam to characterize surface crystallinity during deposition.^12^ During growth, both the specularly reflected and diffracted electrons are imaged on a phosphor screen using a charge-coupled device (CCD) camera. Surface crystallinity and film thickness can be measured by tracking the intensity fluctuations of the reflection or diffraction spots using a mean kernel. In nucleation-limited growth modes, oscillations of the RHEED intensity indicate ordering and disordering processes associated with monolayer formation. Using a mean kernel to average the intensity of a single spot decimates the information. There are essential insights remain unextracted. For example, information about covariance between spot intensities, peak sharpness, and orientation is not considered. There have been a few reports where more insights were extracted from RHEED. Research has demonstrated that RHEED intensity provides valuable insights into the growth mode and can be utilized to extract and analyze surface diffusion kinetics.^12−14^ This is quantified through a characteristic time, primarily determined by the time required for adatoms to stabilize on the surface. The time scales associated with these experiments, however, are on the order of tens of seconds and are, thus, severely limited in applicability. Alternatively, analysis of Kikuchi lines has revealed insight into the inner potential related to the surface dipole and electrostatic potential, allowing the determination of surface chemistry.^15^ To extract more insight from RHEED, researchers have used statistical approaches under the guise of machine learning.^16,17^ For example, principal component analysis (PCA) and *K*-means clustering methods have been used to analyze RHEED recordings to provide insight into surface dynamics and growth-process mechanisms.^16,17^ This has been extended to a phase-mapping method developed to analyze Fe_*x*_O_*y*_ thin-film growth at various temperatures and oxygen pressures.^18^ While machine learning has significant potential, the current approaches are not constrained to physics, making interpretability challenging.^18^ Additionally, machine-learning capabilities could be enhanced by improving the spatial and temporal resolution of the data.^17^

While these reports provide direction for the field, it is still an open challenge to conduct RHEED with sufficient temporal resolution for *in situ* characterization without modification of the growth rates. The cameras, computing infrastructure, and algorithms are the primary challenges with this. Cameras used for RHEED generally record at video rates (60–120 Hz), much slower than the growth kinetics.^19,20^ While high-speed (>500 Hz) streaming cameras are commercially available, saving and processing data collected at upwards of 20 Gbps is challenging on the laboratory scale. For reference, most universities only have 1 Gbps of networking in lab endpoints.

Real-time RHEED monitoring could provide a critical understanding of growth mechanisms and kinetics. For example, RHEED intensity oscillations due to laser ablation can be fit with an exponential function  to interpret how adatoms diffuse, nucleate, and crystallize.^19,21^ Understanding how growth conditions such as temperature and environmental pressure modify and optimize the growth process would then be possible, adding significant value to PLD experiments. For example, the characteristic time τ provides insight into the activation energy, surface diffusion, growth modes, and surface termination.^13,20,22^ This insight, particularly if the analysis were conducted automatically and in real-time, could be used to control growth processes and enable the synthesis of more complex emerging heterostructure designs, including superlattice,^23,24^ defect-graded,^25^ and compositionally graded structures.^26^

Here, we design a platform to explore growth kinetics in PLD using high-speed RHEED at >500 Hz. Using the homoepitaxy of a model perovskite, SrTiO_3_, we explore how substrate miscut and surface termination affect per-plume diffusion processes and growth kinetics. We develop an open-source analysis package for fitting RHEED images to a 2D Gaussian distribution, allowing the extraction of amplitude, orientation, and characteristics of diffuse scattering. We observe different per-plume RHEED intensity decay associated with surface dynamics and the transitions between layer-by-layer and mixed growth modes (island growth and layer-by-layer). These are identified as inversions in surface dynamics related to the surface termination, which drives attractive or repulsive surface-diffusion processes. Additionally, we observe that the characteristic time is highly dependent on the substrate miscut and, in turn, the step width. Finally, by monitoring changes in RHEED kinetics, we can better predict half-monolayer completion. Ultimately, this work demonstrates a new infrastructure for high-speed monitoring of surface kinetics in RHEED. The extension and widespread use of high-speed RHEED could significantly increase the fecundity of nonequilibrium epitaxy and heteroepitaxy.

## Result and Discussion

Homoepitaxy of (001)-SrTiO_3_ using PLD represents a model epitaxial system known to grow in a Frank–Van der Merwe or layer-by-layer growth mode.^27,28^ Layer-by-layer growth occurs when the surface cohesive force is higher than the cohesive force between adatoms. Adatoms form nuclei or diffuse to existing nuclei or step edges. Since the adatoms’ interaction is more substantial with the surface than with each other, they do not form three-dimensional (3D) islands. The simplicity of growth makes it ideal for fundamental studies of growth kinetics. Previous studies have identified many factors that affect growth kinetics, including temperature, pressure, substrate miscut, and surface termination.^4,21,27,29^

Before deposition, tapping-mode atomic force microscopy (AFM) images of (001)-oriented SrTiO_3_ substrates were acquired and used to preselect substrates with different miscut angles and, thus, atomic step widths. Two of these substrates were then selectively treated to be TiO_2_ terminated using a buffered HF-etch process (Methods). Images of the substrates before deposition are provided (Figure S1). Homoepitaxial films of (001)-oriented SrTiO_3_ were deposited using PLD (Methods), where the deposition was monitored using our high-speed RHEED method. Different samples of (001)-oriented SrTiO_3_ were studied as a model system: (i) A TiO_2_-terminated crystal with wide step widths of 213 ± 88 nm and a miscut angle of 0.131°, hereafter referred to as **treated_213 nm −0.131°**; (ii) a TiO_2_-terminated crystal with a narrow step width of 81 ± 44 nm and a miscut of 0.330°, **treated_81 nm −0.330°**; (iii) an untreated, mixed-terminated (SrO and TiO_2_) crystal with a moderate step width of 162 ± 83 nm and a miscut of 0.090°, **untreated_162 nm −0.090°**.

**Figure 1:**
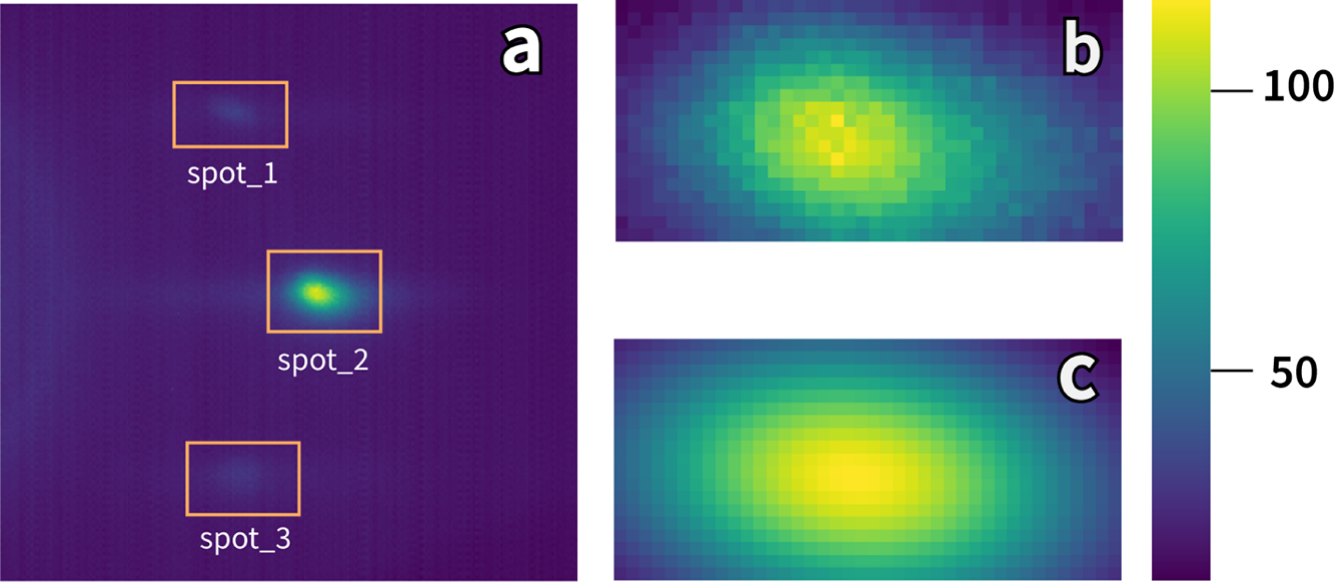
Sample image of RHEED diffraction spot captured during growth. Note that the direct beam is cropped to emphasize the diffraction spots. (a) Image captured by the high-speed camera during the PLD experiment, with three spots labeled as spot 1, spot 2, and spot 3. (b) Cropped region of spot 2 from the full-view image. (c) Regenerated image with 2D Gaussian fit parameters.

All depositions were conducted under identical conditions with a substrate heater temperature of 660 °C in a 50 mTorr partial pressure of oxygen. Targets (Single Crystal SrTiO_3_, Crystec GmbH) were ablated with a 248 nm KrF excimer laser at a fluence of 1.8 J/cm^2^ and a spot size of ∼1.6 mm × 4.7 mm. RHEED was continuously imaged at 500 Hz or frames per second using a Phantom Vision S210 streaming camera (Methods) at a resolution of 1024 × 1280 pixels. A representative diffraction image at this frame rate is provided (Figure 1). The X-ray diffraction and reciprocal space mapping results in (002) and (103) orientations confirm high-quality SrTiO_3_ homoepitaxy film (Supporting Information Figure S2). During RHEED, the electron beam interacts with only the first several unit cells of the thin-film surface due to the grazing incidence angle (1–2°). The diffracted electron beams constructively interfere, generating diffraction spots. We have sufficient signal to observe the zeroth (spot_2 in Figure 1) and first-order diffraction spots (spot_1 and spot_3 in Figure 1). While all these spots can be used for analysis, we selected the zeroth-order diffraction spot as it has the highest signal-to-noise.

**Figure 2:**
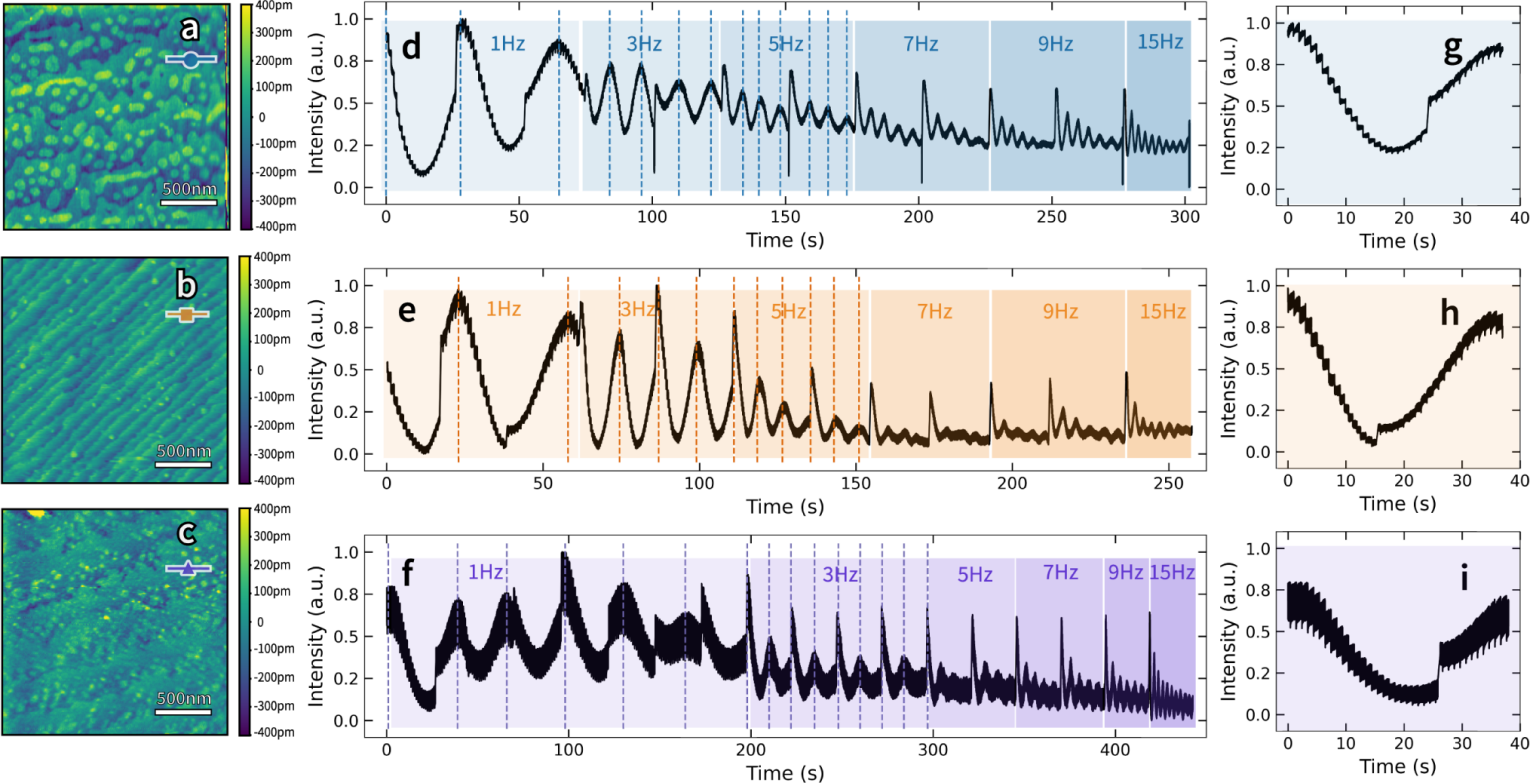
RHEED intensity plots and corresponding AFM images. AFM images of SrTiO_3_ thin film in (a) **treated_213 nm −0.131°**, (b) **treated_81 nm −0.330°**, and (c) **untreated_162 nm −0.090°**, respectively. (d–f) RHEED intensity of spot 2 in different experimental conditions. Dashed lines indicate the growth of one unit cell. (g–i) Partially crop and magnified RHEED intensity scatter plot from three plots (d–f).

Following growth, the surface topography was measured using tapping mode AFM (Figure 2a–c). All samples show surface topography indicative of layer-by-layer growth; however, there are essential differences upon closer inspection. Starting with the treated sample with the largest step width, **treated_213 nm −0.131°** (Figure 2a), we observed well-defined monolayers with some islands/clusters on the steps. The appearance of islands indicates that forming local nuclei at the end of the growth is allowable, rather than diffusing the long distance to the step edge. The **treated_81 nm −0.330°** sample (Figure 2b) reveals much narrower step widths and some evidence of nuclei formation within the steps. Following growth, the topography appears as if it is close to the transition to step bunching.^30^ For the **untreated_162 nm −0.090°** sample (Figure 2c), a similar surface morphology with a higher density of surface nuclei is observed. This can be explained by the large step width and an increased propensity for surface nucleation due to the mixed surface termination.

We calculated each sample’s RHEED intensity by fitting the zeroth-order diffraction spot with a 2D Gaussian (d–f) at 1 to 15 Hz deposition frequencies. The 2D Gaussian equation is
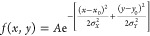
1where *A* is the amplitude, (*x*_0_,*y*_0_) is the spot center position, and (*σ*_*x*_,*σ*_*y*_) is the spread in the *x*, and *y* directions , respectively. The Gaussian fit provides additional parameters as descriptors for the diffraction spot and can be useful for further analysis. However, we primaryly use the sum of the diffraction spot intensity as the descriptor for analysis. The results from other metrics derived from the 2D Gaussian fits are also included (Figures S3–S5). The different background intensities indicate each growth frequency, as labeled. These images show intensity fluctuations associated with ordering and disordering during monolayer formation, typical of layer-by-layer growth. Beyond this point, acquiring RHEED images at high frame rates allows subtleties in the intensity of the diffraction spots to be observed. By magnifying the temporal resolution of the RHEED intensity of the zeroth-order diffraction spot, we observe exponential variations in intensity synchronized with each laser pulse. These intensity oscillations indicate intrapulse surface ordering processes, usually outside the temporal resolution of conventional RHEED imaging. Similar phenomena were observed using slower-than-practical deposition rates (0.5–5 Hz) with conventional 60 Hz RHEED systems.^21,31^ We extracted the temperature-related RHEED intensity and characteristic time by fitting the RHEED intensity decay.

**Figure 3:**
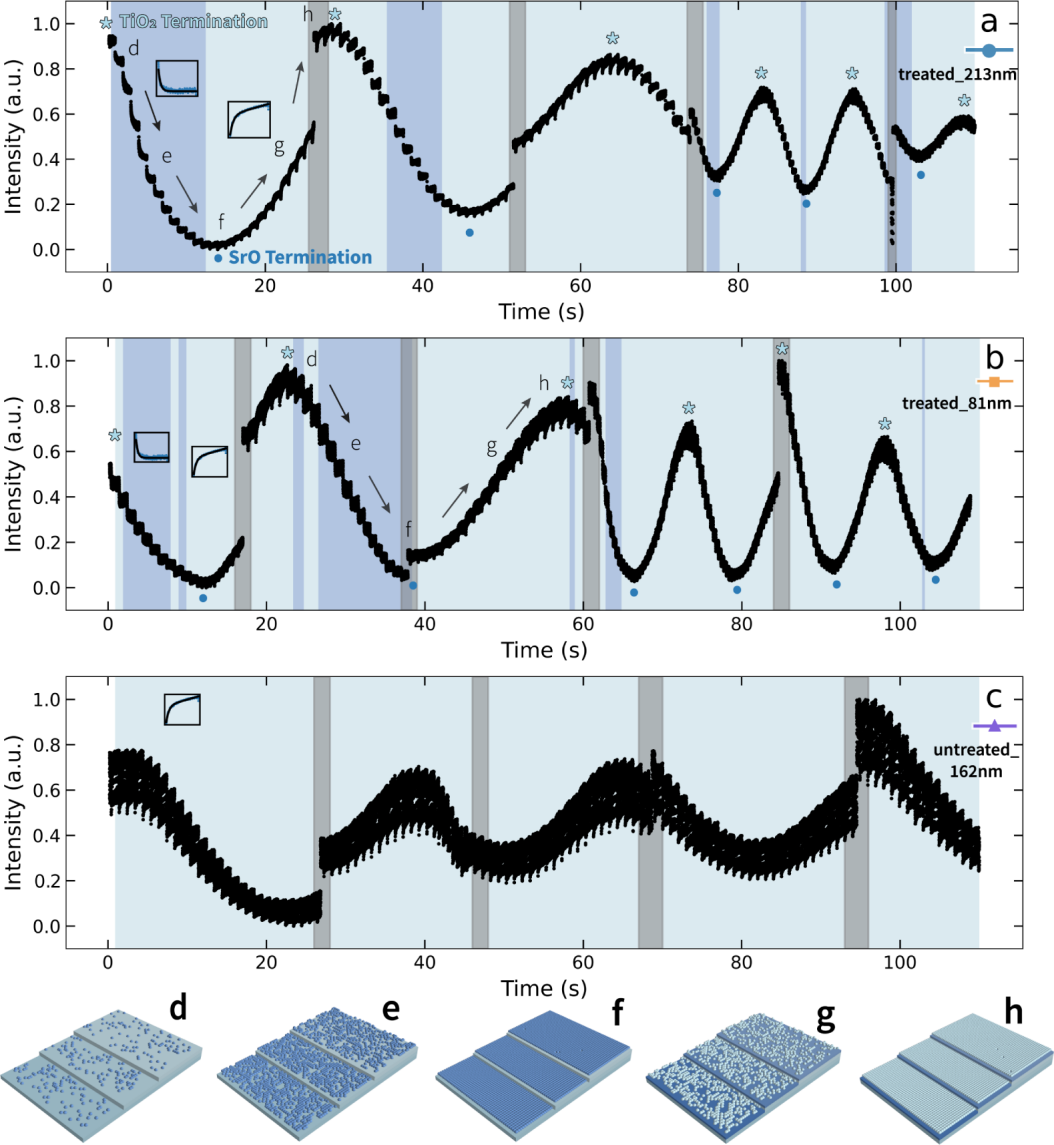
Temporal evolution of RHEED intensity extracted from the spot 2 region. RHEED intensity during deposition of (a) **treated_213 nm** −**0.131°**, (b) **treated_81 nm** −**0.330°**, and (c) **untreated_162 nm** −**0.090°,** respectively. The gray background indicates changing growth conditions, while dark and light backgrounds represent different fitting functions for the decay curves. Typical decay curves are provided on their corresponding background colors. We labeled the schematic drawings of the surface structure during PLD, (d) initial deposition on a monolayer, (e) continued deposition, (f) half-monolayer with either nucleus forming at the step edge or on the surface, (g) continuation of monolayer growth, (h) near-completion of monolayer growth. In treated substrate samples, the surface will go through (d–f) when intensity decreases and from (f–h) when intensity increases.

These intrapulse intensity fluctuations can be explained by considering the deposition process. Each laser pulse forms a plasma plume that deposits adatoms on the substrate surface. These high-energy adatoms diffuse along the surface until they form islands or attach to the step edges as part of a new layer. Depending on the instantaneous surface structure, atoms can increase the surface disorder by introducing more clusters/islands or increase the surface order by linking existing clusters/islands, resulting in an increase or decrease decaying in the RHEED intensity, respectively, aligning with every laser pulse. In addition to diffraction intensity being affected by surface ordering, it has been reported that different surface terminations can exhibit different diffraction intensities due to the different surface energies between TiO_2_-terminated surfaces and SrO-terminated surfaces.^15,32^ By controlling the electron beam’s incident angle, we can control which termination or sublayer corresponds to the oscillation peak or valley. As deposition starts from a TiO_2_-terminated surface for treated substrates, the oscillation peak corresponds to the TiO_2_-terminated surface and the oscillation valley corresponds to the SrO-terminated surface, supported by the observations in Figures 2 and 3.

There are evident differences in the intraplume RHEED intensity throughout the growth process that change with initial substrate termination (Figure 3a,b). To extract more insight, we fit the dynamic intensity oscillation by detecting each pulse using *the scipy.signal.find_peaks* function in Python. We normalize the RHEED intensity by dividing it by the maximum intensity [*s.t.*, the intensity is (0,1)]. The recovery curve was automatically fitted to two exponential functions (eqs 2 and 3)
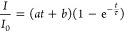
2
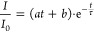
3depending on the sign of the trend. Here, ***I*** is the RHEED intensity, *I*_0_ is the saturation intensity, *t* is the diffusion time, which starts from the instant of laser incident, *τ* is the characteristic time, and  is the linear correction for the intensity magnitude. We selected the appropriate fitting function with the lowest mean squared error (MSE) in each intrapulse region. These two functions describe different growth regimes, where the first represents exponential decay (i.e., decaying to a minimum value), while the second represents exponential stabilization (i.e., exponentially increasing to a maximum value). These two processes indicate that the surface kinetics evolve to a more disordered (ordered) state, respectively, with adatom surface diffusions following deposition. The fitting results of the three samples’ first several growth periods are shown (Figure 3). Additional details of the fitting process and results are provided in Figures S9–S11.

Depending on the surface state, different mechanisms are observed. When the substrates are TiO_2_-terminated, we observe the exponential function flipping at the valleys and peaks (Figure 3a,b). However, only the stabilization process is observed in the untreated substrates (Figure3c). We hypothesize that on TiO_2_-terminated substrates, adatoms will diffuse to form monolayer clusters when growing toward the SrO sublayer (with the dark-blue background, Figure 3). Here, the forces between adatoms are essentially repulsive, resulting in a more disordered surface and the exponential decay of RHEED intensity. Once the SrO sublayer is formed, the behavior changes to exponential stabilization, where new adatoms experience a predominantly attractive force with the surface (with the light background, Figure 3) leading to an exponential increase in intensity. At the transition between these two phenomena, neither model can be applied. When the process is modeled by an exponential decay, the bonding forces with the surface are relatively low, requiring significant diffusion to discover stable binding sites. Conversely, the stabilization response indicates strong, attractive forces with the surface. The attractive forces could either result from strong bonding with the surface or a transition to an island mode. First-principles calculations of (001) TiO_2_-terminated surfaces generally have lower surface energy than SrO-terminated surfaces.^32^ Thus, consistent with our observations, the adatom attraction when growing the TiO_2_ sublayer is stronger as it decreases the surface energy.

As the growth progresses, we observe that the intensity amplitude generally decreases with deposition, implying a partial transition from 2D growth to 3D growth. We note some increases in intensity associated with additional surface reconstruction due to delays in deposition when changing growth frequencies. There is also a correlated decrease in the Gaussian-fit parameters—2D height and width—of the RHEED images (Figures S3–S5). This change relates to a transition from streaks to spots, commonly associated with 2D-to-3D growth modes, and coincides with a suppression of the “flipping phenomenon,” where the model alternates from exponential decay to exponential stabilization. We conjecture that when more islands form, adatom bonding becomes easier, resulting in exponential stabilization in the RHEED intensity. Eventually, only the stabilization phenomenon is observed.

In the untreated substrate, purely exponential stabilization phenomena are observed. This implies that the adatoms experience a much stronger attractive force with the surface and transition more quickly to 3D growth. This is substantiated by AFM (Figure 2c), where the surface topography reveals small, island-like structures. This also coincides with a decrease in the intensity of the RHEED peaks and is associated with the onset of a transition from streaks to spots, further indicating the transition from 2D to 3D growth (Figure S6).

**Figure 4:**
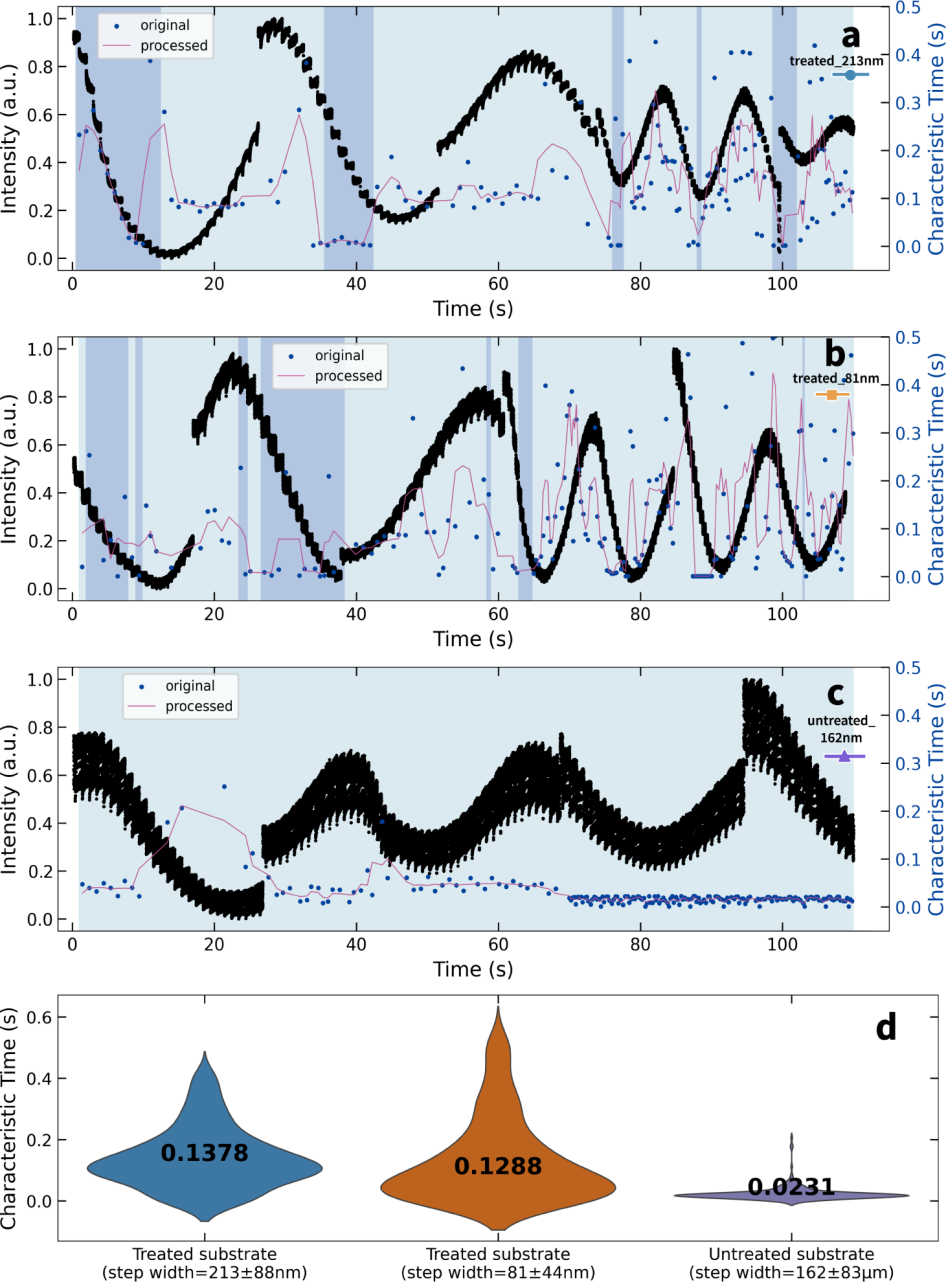
RHEED intensity and characteristic time of (a) **treated_213 nm −0.131°**, (b) **treated_81 nm −0.330°**, (c) **untreated_162 nm −0.090°**, respectively. The black scatter, blue scatter, and red line plots are RHEED intensity, fitted characteristic time, and processed characteristic time, accordingly. Note that we normalize the raw characteristic time results (blue points) so we have the processed characteristic time (red curved), as described in the source code. The dark blue and light gray backgrounds indicate different fitting functions (Figure 3d) Violin plot of average characteristic time of three samples.

To explore the effects of miscut and surface termination on growth kinetics, we summarize the characteristic time ***τ*** for zeroth-order diffraction spots (Figure 1). The characteristic time describes the time required for the influx of deposited atoms to reach a steady state. The calculated characteristic times are τ = 0.1366, 0.1288, and 0.0245 s for the treated_213 nm −0.131°, treated_81 nm −0.330°, and untreated_162 nm −0.090° samples, respectively. This indicates, in line with expectations, that reduced step width and mixed substrate termination decrease the time adatoms are remain actively diffusing. To further quantify the evolution of the characteristic time and its distributions, we plot the temporal evolution of the characteristic time (*τ*, red) superimposed on the RHEED intensity (Figure 4a–c). This provides insights into the surface kinetics during deposition. In the sample with the widest crystallographic steps (treated_213 nm −0.131°, Figure 4a), we observe oscillations in the characteristic time, which are maximized at the half-monolayer (TiO_2_- or SrO terminated surface), where diffusion distances to nucleation sites are maximized.

Furthermore, there is evidence that the characteristic time is longer when growing half-monolayers (transition from TiO_2_ termination to SrO termination) than the TiO_2_ sublayer (transit from SrO termination to TiO_2_ termination). Once again, this can be attributed to the lower surface energy of TiO_2_-terminated surfaces. Moving on to treated_81 nm −0.330° (Figure 4b), we observe an overall reduction in the characteristic times, as expected with the decreased diffusion distance associated with narrower step widths. Similarly, we observe a maximum at the half-monolayer/full sublayer (clean single termination surface); however, this is less pronounced as the characteristic time is dominated by adatom diffusion to the step edge. Finally, untreated_162 nm −0.090° exhibits the shortest characteristic time, indicating a greater number of binding sites because of the mixed termination (Figure 4c). Similarly, the characteristic times reach a maximum at the half-monolayers (clean single termination surface), coinciding with minimum intensity. This suggests that the initial substrate was preferentially TiO_2_ terminated, as expected, due to the lower surface energy. The nature of the surface termination at the peaks (minima) of the RHEED intensity cannot be determined unambiguously from our experiments, as the relative intensity of the surface terminations can vary (flip) depending on the incidence angle based on the “flipping” phenomenon.^15^ These results demonstrate that increased RHEED temporal resolution provides additional insight into growth kinetics, extending well beyond the Nyquist frequency of surface kinetics and nearly an order of magnitude faster than traditional RHEED systems.

## Conclusion

We developed a high-speed RHEED platform to increase imaging rates beyond 500 Hz, allowing the direct observation of surface reconstruction kinetics in PLD. Using homoepitaxial growth of SrTiO_3_ as a model system, we developed an open-source, automated, and reproducible RHEED analysis pipeline to investigate the temporal evolution of the RHEED intensity and eccentricity. By extracting the characteristic time of surface reconstruction, we quantified the dynamics of surface adsorption and reconstruction associated with diffusion distance to steps/nuclei sites and the binding energy of differently terminated surfaces. Specifically, we observe decreased characteristic times with mixed-surface termination, decreased step-width, and SrO-termination, where diffusion distances are reduced or surface binding is stronger. This work motivates the application of high-speed RHEED in PLD of more complex material systems. In future work, we aim to extend this technique to other systems, such as SrRuO_3_ or BaTiO_3_, to utilize this platform for deeper insights into deposition dynamics. This increased temporal resolution provided by high-speed RHEED also provides a fertile playground for developing machine-learning techniques to extract statistical insights into growth dynamics. With advanced computing hardware and control systems, high-speed RHEED could serve as an invaluable tool to compensate for exogenous changes that alter growth processes during PLD. This work is a step toward future integrated control systems leveraging high-speed RHEED, which could increase the fecundity of PLD and enable the design of complex heterostructures and interfaces that are unattainable using manual control systems. Furthermore, these concepts could be applied to improve the temporal resolution of other *in situ* diagnostics for monitoring additive manufacturing processes.

## Methods

Homoepitaxial SrTiO_3_ thin films were grown from single crystal SrTiO_3_ (001) targets on SrTiO_3_ (001) substrates by PLD at 660 °C in a background pressure of 50 mTorr O_2_. Following growth, samples were cooled to room temperature in 30 Torr O_2_. Some SrTiO_3_ substrates were treated with buffered HF solution (NH_4_F 30–50%, HF 1–5%) followed by a 3 h annealing process at 1000 °C in an oxygen environment. The substrate step width was determined by 1. detecting the peaks (step edge) on the *x*/*y*–*z* profile plot with the scipy.signal.find_peaks function; 2. calculating the distance between nearby peaks; 3. Correcting the step height with the theoretical value of −0.391 nm for one unit cell—since the measured step height can be biased by AFM measurement, especially for the narrow step width. Surface morphology (atomic force microscopy) is characterized by an Oxford Instrument MFP-3D Atomic Force Microscope. The crystallography analysis (X-ray Diffraction and Reciprocal Space Mapping) was conducted with Dr. Liyan Wu on a Panalytical Empyrean X-ray diffractometer.

Due to the coupling effect of the substrate heater and electron diffraction, electron diffraction will interfere with the electric field generated by the heater of the substrate holder; we suspend the substrate heater during the experiment to minimize the interaction between the sample heater and the electron beam. However, such an operation results in a 15 °C decrease in temperature in every growth period (50–60 s). Since all films were grown under the same temperature conditions, the assumption has been made that the growth temperature will not be a relevant factor contributing to the variation of the growth dynamics.

The images are collected by a Phantom Machine Vision S210 camera and streamed using an Euresys Coaxlink Quad CXP-6 video acquisition card and Euresys eGrabber image acquisition software. The camera acquisition speed is 500 Hz, or frames per second, at a resolution of 1024 × 1280 pixels. The acquired RHEED intensity data is analyzed with the following steps:

1. Crop the full-view image containing multiple diffraction spots into different regions.
2. Fit the RHEED spot with a 2D Gaussian function for metrics such as sum and maximum values.
3. Construct time-dependent RHEED intensity metrics for different spots and metrics to determine RHEED oscillation.
4. Use *scipy.signal.find_peaks* and customized data processing functions in Python to separate RHEED intensity curves for every laser ablation.
5. Fit the RHEED curves with *the scipy.optimize.curve_fit* function and customized functions in Python for the characteristic time.

All analyses are available as reproducible JupyterBook.

## Data Availability

All data and reproducible code are made openly available under the BSD-2 License. The full dataset release is available on Zenodo via DOI 10.5281/zenodo.7934968, with a compressed version of the data available at DOI 10.5281/zenodo.7948591. To improve accessibility, a Jupyter Book is available at https://m3-learning.github.io/Predicting-Pulsed-Laser-Deposition-SrTiO3-Homoepitaxy-Growth-Dynamics-using-RHEED/content/index.html.

## Supporting Information Available

**Figure S1,** AFM images for substrates; **Figure S2,**X-ray Diffraction result for a typical SrTiO_3_ substrate and three thin films; **Figure S3,**Gaussian fitting results for RHEED spot images of sample treated_213 nm; **Figure S4,**Gaussian fitting results for RHEED spot images of sample treated_81 nm; **Figure S5,**Gaussian fitting results for RHEED spot images of sample untreated_162 nm; **Figure S6,**RHEED pattern for sample untreated_162 nm at *t* = 0 s and *t* = 22 s, respectively; **Figure S7,**unprocessed magnitude **a**, **b** and characteristic time τ for decay curves of sample treated_213 nm; **Figure S8,**unprocessed magnitude **a**, **b** and characteristic time τ for decay curves of sample treated_213 nm; **Figure S9**, unprocessed magnitude **a**, **b** and characteristic time τ for decay curves of sample treated_213 nm; **Figure S10**fitting process with raw data, prediction curve, and failed prediction curve for sample treated_213 nm; **Figure S11,**fitting process with raw data, prediction curve, and failed prediction curve for sample treated_162 nm; **Figure S12,**fitting process with raw data, prediction curve and failed prediction curve for sample untreated_81 nm (PDF)
