# Supplementary material for: Predicting Pulsed-Laser Deposition SrTiO3 Homoepitaxy Growth Dynamics Using High-Speed Reflection High-Energy Electron Diffraction
Source: ACS Appl Mater Interfaces. 2025 Apr 8;17(16):24485–93. doi: 10.1021/acsami.4c12655 (PMC12022940; doi:10.1021/acsami.4c12655)
Supplement: Supplementary file 1 — am4c12655_si_001.pdf [file am4c12655_si_001.pdf]

Predicting Pulsed-Laser Deposition $SrTiO_3$ Homoepitaxy Growth Dynamics using High-Speed Reflection High-Energy Electron Diffraction

Supporting Information

Yichen Guo[†,1,2], Peter Meisenheimer[†,3], Shuyu Qin[4], Xinqiao Zhang[2], Julian Goddy[2], Ramamoorthy Ramesh[5,6], Lane W. Martin[3,7], Joshua Agar[2,*]

[†] Authors contributed equally to this work

[1] Department of Materials Science and Engineering, Lehigh University, Bethlehem, PA 18015, USA

[2] Department of Mechanical Engineering and Mechanics, Drexel University, Philadelphia, PA 19104, USA

[3] Department of Materials Science and Engineering, University of California, Berkeley, CA 94720, USA

[4] Department of Computer Science and Engineering, Lehigh University, Bethlehem, PA 18015, USA

[5] Department of Materials Science and Nanoengineering, Rice University, Houston, TX 77005, USA

[6] Department of Physics and Astronomy, Rice University, Houston, TX 77005, USA

[7] Materials Sciences Division, Lawrence Berkeley National Laboratory, Berkeley, California 94720, USA

[*] Email address: jca92@drexel.edu

Supporting Figures

1. AFM Images

Prior to growth, substrates were prescreened using tapping mode AFM. We selected substrates received from the supplier with slightly different miscuts for our studies (**Figure S1**). The step width was calculated using a script provided in the Jupyter Notebook. From the images, we can see atomically sharp steps in all substrates. The treated substrates show cleaner step edges and surfaces than the untreated substrate. This is consistent with prior reports.

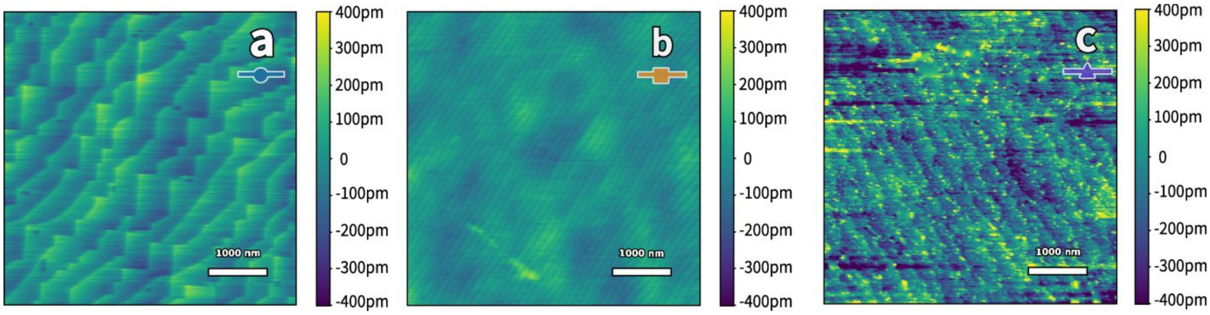

Figure S1 AFM images for substrates. **a** Substrate AFM image for sample treated_213nm. **b** Substrate AFM image for sample treated_81nm. **c** Substrate AFM image for sample untreated_162nm.

2. X-Ray Diffraction Studies

Following growth, θ-2θ scan and symmetric and asymmetric reciprocal space maps were conducted (**Figure S2**). The line scans symmetric and Asymmetric reciprocal space maps show a single prominent diffraction peak. There was no secondary diffraction peaks identified, and the peaks were not found to be significantly broader than the substrate. This implies that all the growth was of high quality, as would be expected for homoepitaxial growth of $SrTiO_3$.

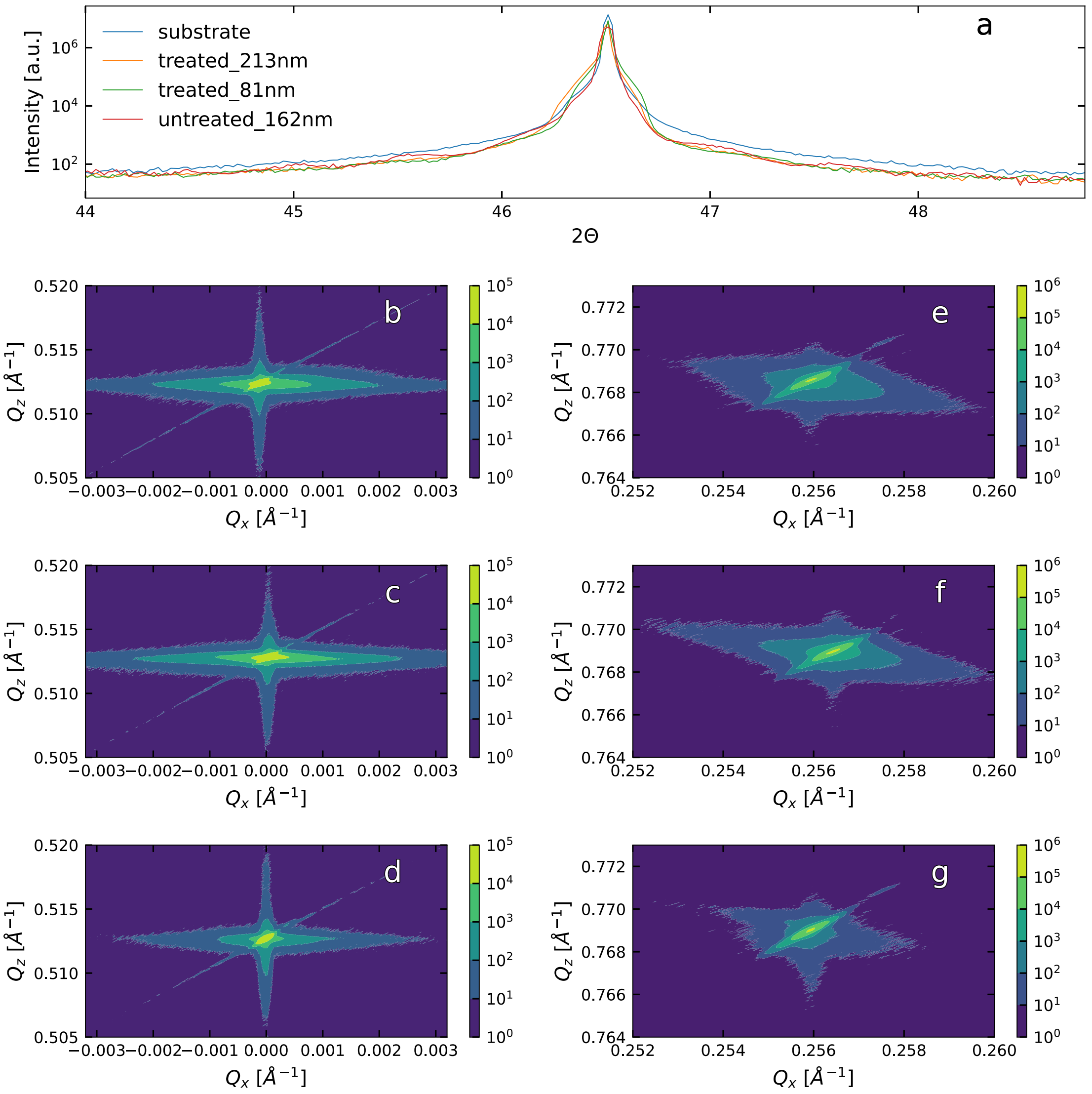

Figure S2 X-ray Diffraction result for a typical $SrTiO_3$ substrate and three thin films. **b, c, d** Reciprocal Space Mapping results in (002) orientation for sample treated_213nm, treated_81nm and untreated_162nm, respectively. **e, f, g** Reciprocal Space Mapping results in (103) orientation for sample treated_213nm, treated_81nm and untreated_162nm, respectively.

3. Further Analysis details

For completeness, we provide the temporal evolution of all Gaussian fit parameters (**Figure S3**, **Figure S4**, **Figure S5**) for the samples studied. Most of the parameters have a high covariance with the intensity during growth and thus provide an indication of monolayer formation. A snapshot of RHEED spots in different deposition time is exhibited in **Figure S6** to act as reference for analysis.

We further provide the full details from the exponential fits (**Figure S7**, **Figure S8**, **Figure S9**); from these figures, we can see that as the intensity, and thus surface quality, decreases with growth progression, the direct observation of surface reconstruction becomes challenging. Finally, we show the RHEED intensity and fit results for each pulse at 1 Hz (**Figure S10**, **Figure S11**, **Figure S12**). We highlight the

selected and failed model in each figure. We note that further details are best explored interactively in the provided source code.

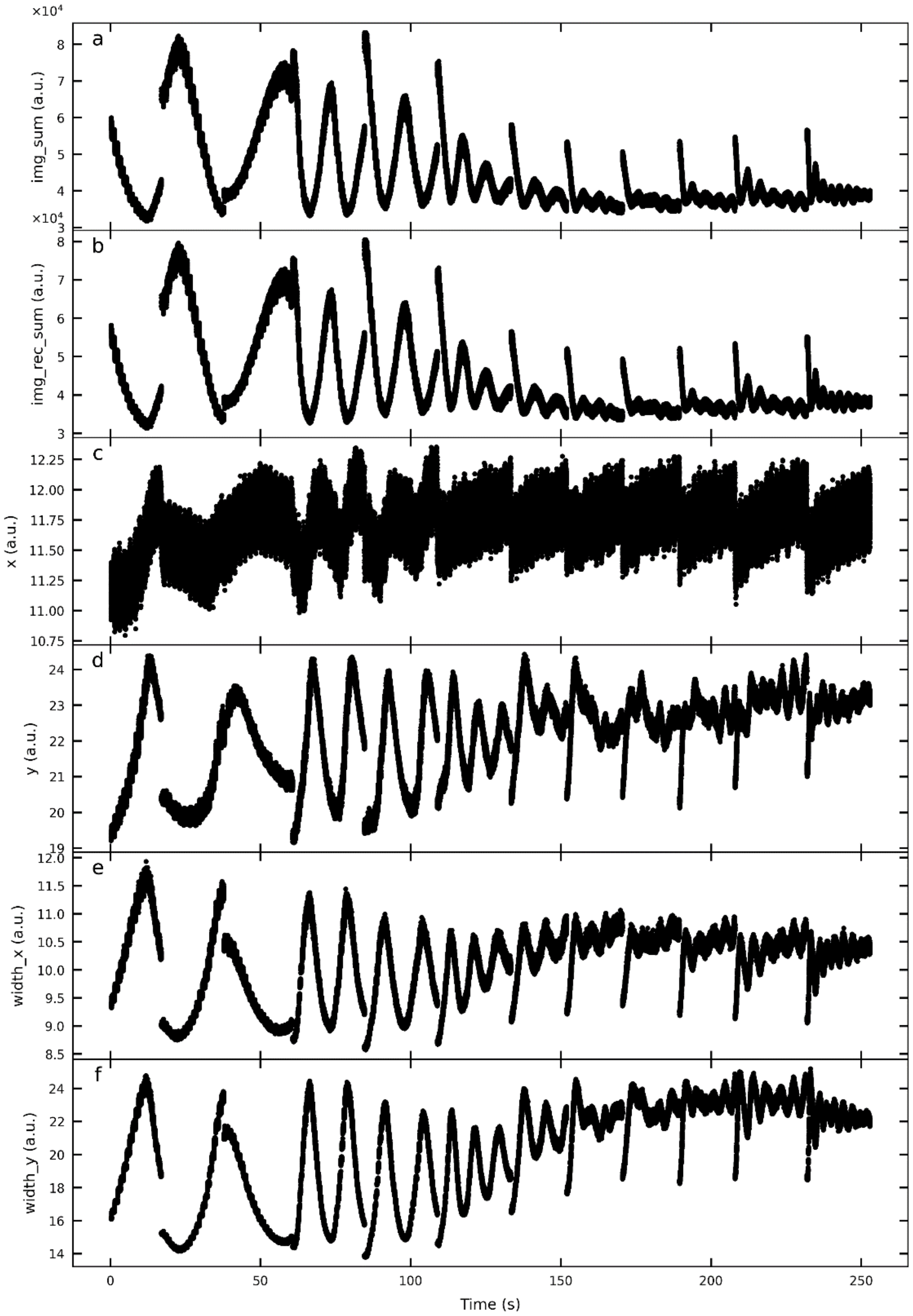

Figure S3 Gaussian fitting results for RHEED spot images of sample treated_213nm. **a** Intensity sum of original image. **b** Intensity sum of reconstructed image, **c** Spot center in spot x coordinate. **d** Spot center in y coordinate. **e** Spot width in x coordinate. **f** Spot width in y coordinate.

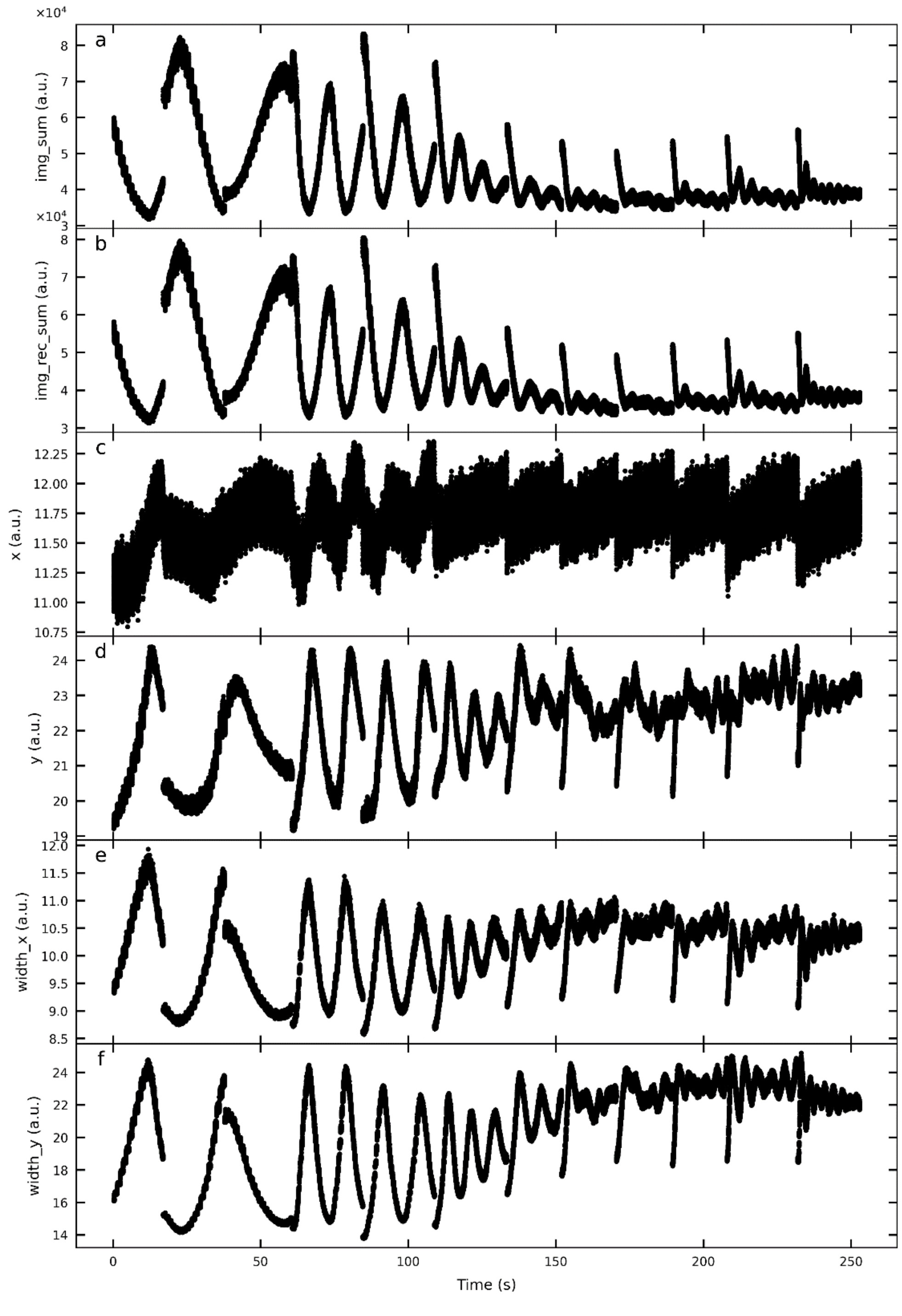

Figure S4 Gaussian fitting results for RHEED spot images of sample treated_81nm. **a** Intensity sum of original image. **b** Intensity sum of reconstructed image, **c** Spot center in spot x coordinate. **d** Spot center in y coordinate. **e** Spot width in x coordinate. **f** Spot width in y coordinate.

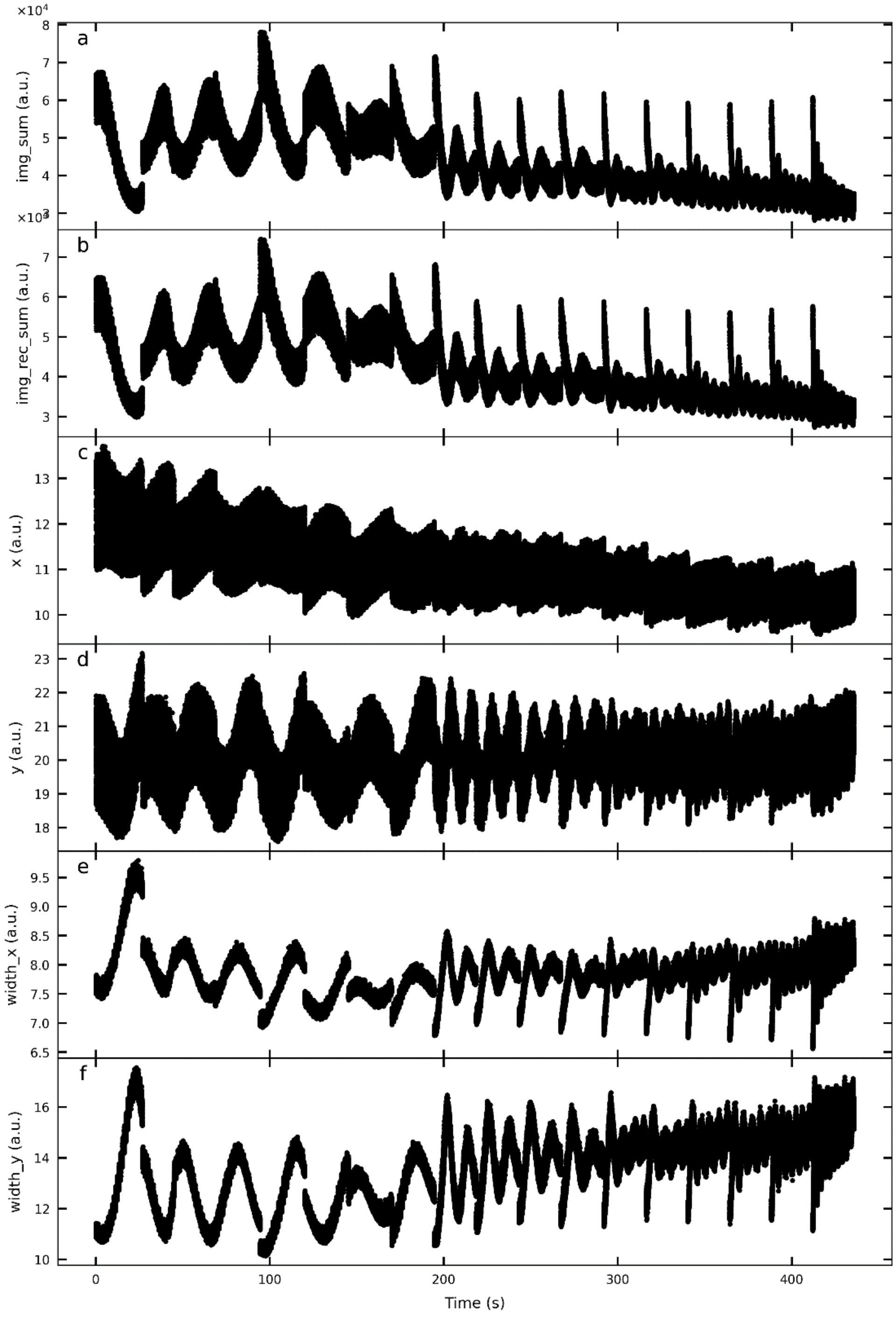

Figure S5 Gaussian fitting results for RHEED spot images of sample untreated_162nm. **a** Intensity sum of original image. **b** Intensity sum of reconstructed image, **c** Spot center's x position in the x, y coordinate. **d** Spot center's y position in the x, y coordinate. **e** Spot width in x coordinate. **f** Spot width in y coordinate.

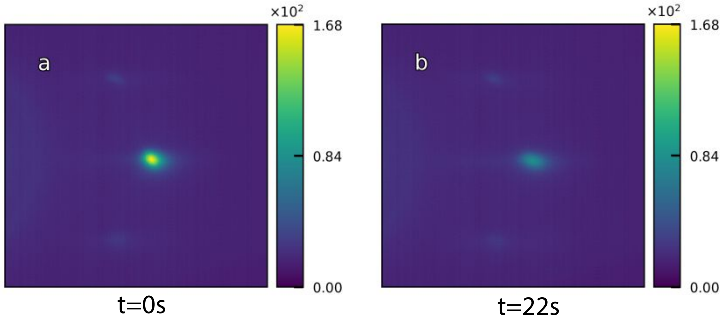

Figure S6 a, b RHEED pattern for sample untreated_162nm at t=0s and t=22s, respectively.

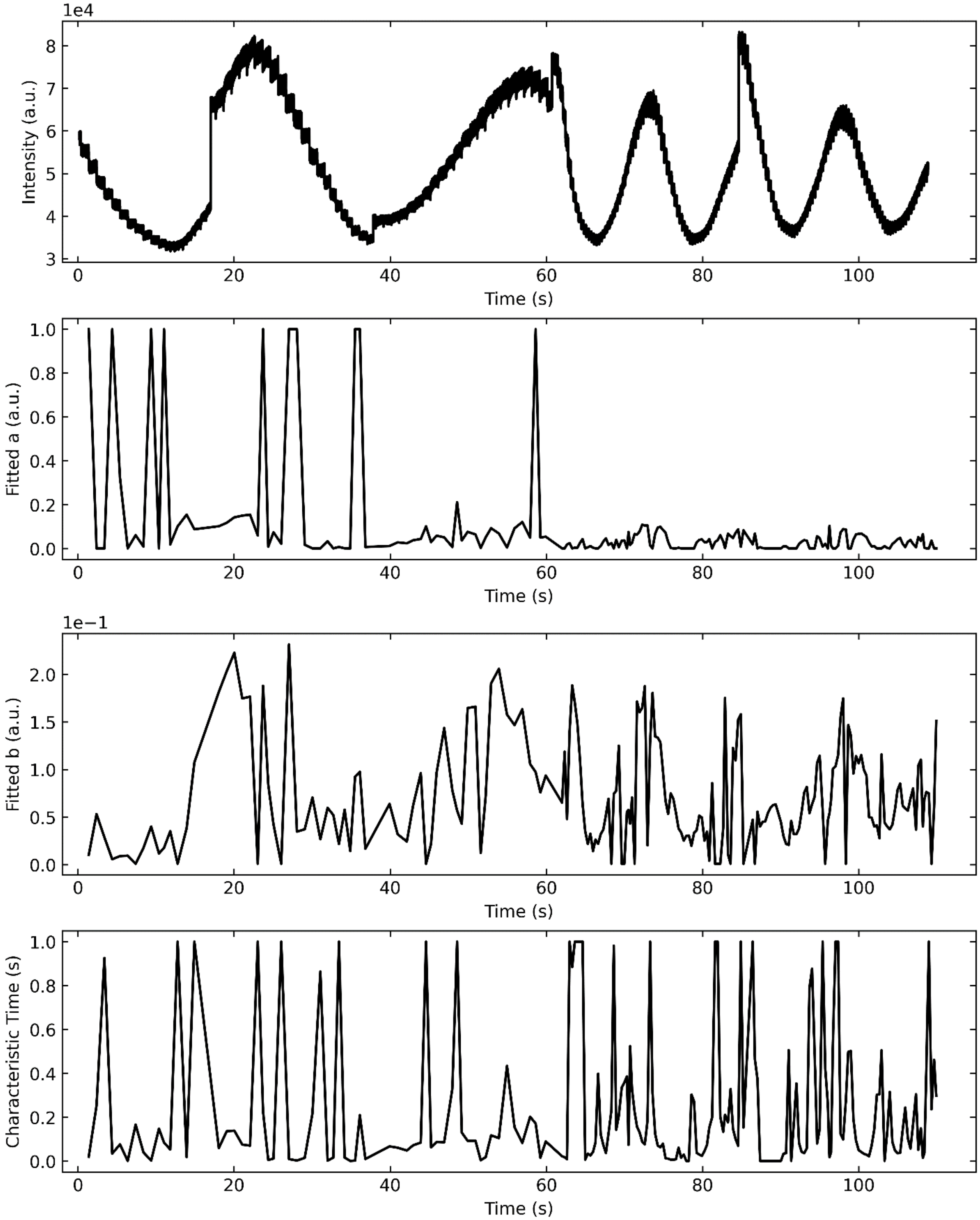

Figure S7 Unprocessed magnitude **a**, **b** and characteristic time τ for decay curves of sample treated_213nm.

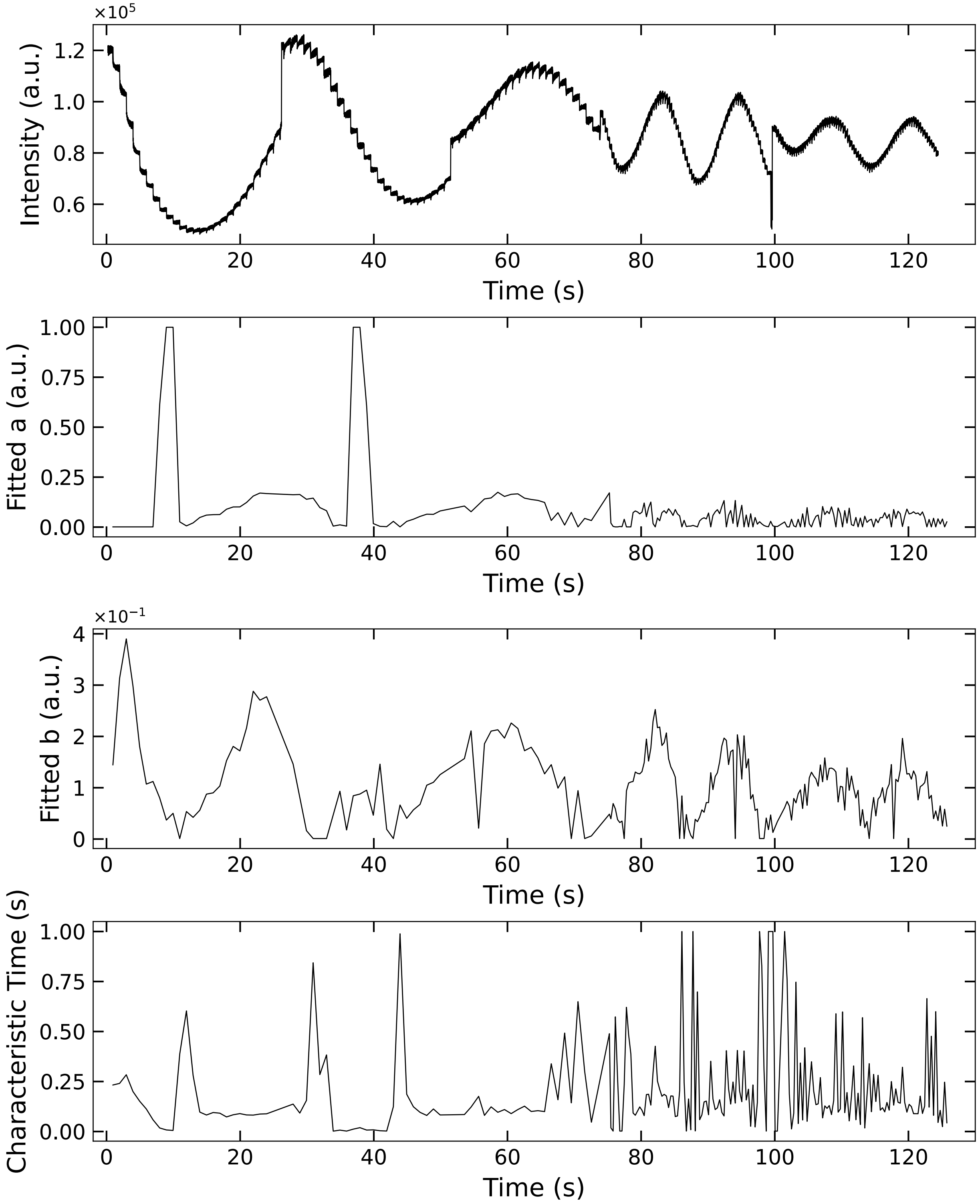

Figure S8 Unprocessed magnitude **a**, **b** and characteristic time τ for decay curves of sample treated_213nm.

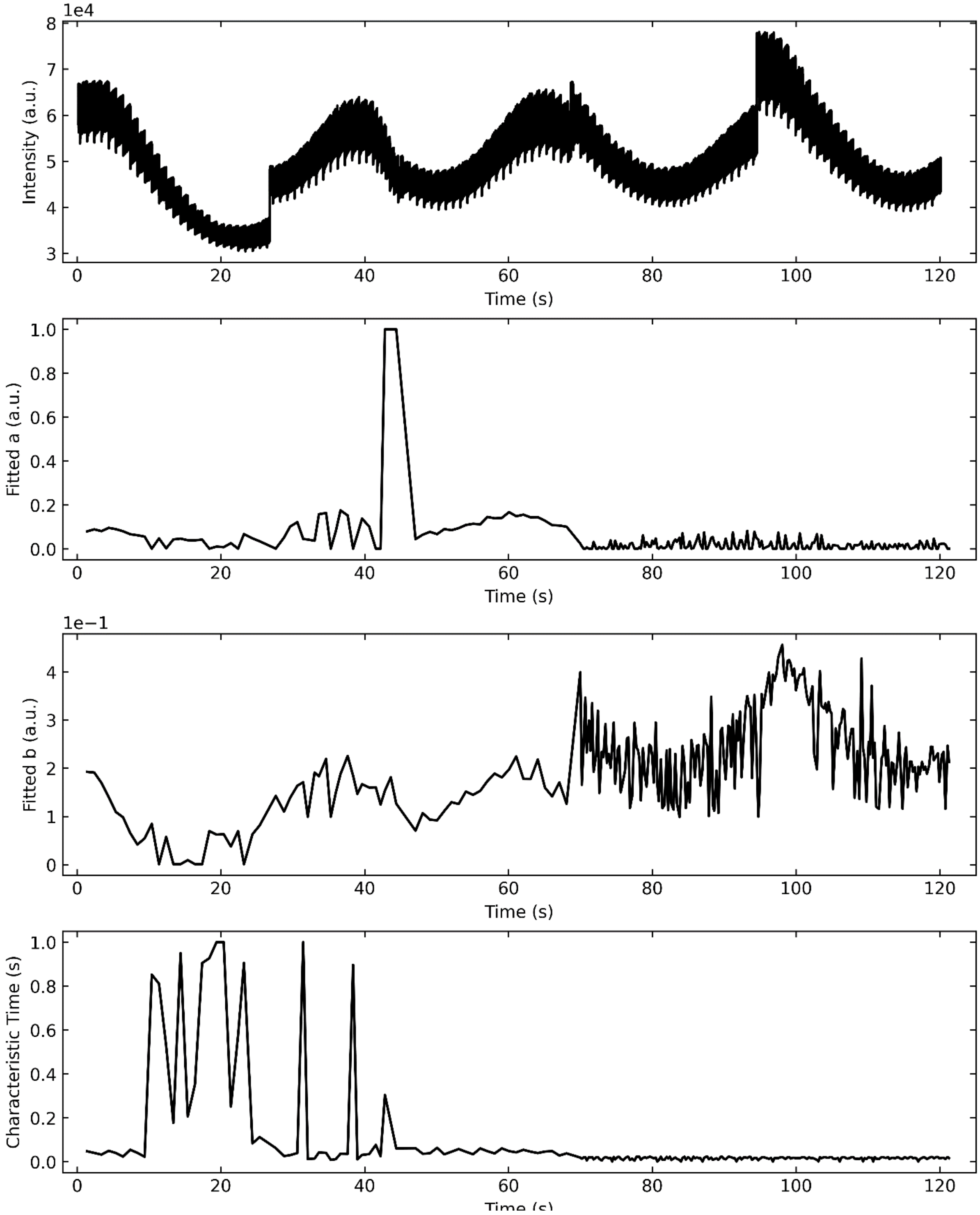

Figure S9 Unprocessed magnitude **a**, **b** and characteristic time τ for decay curves of sample untreated_162nm.

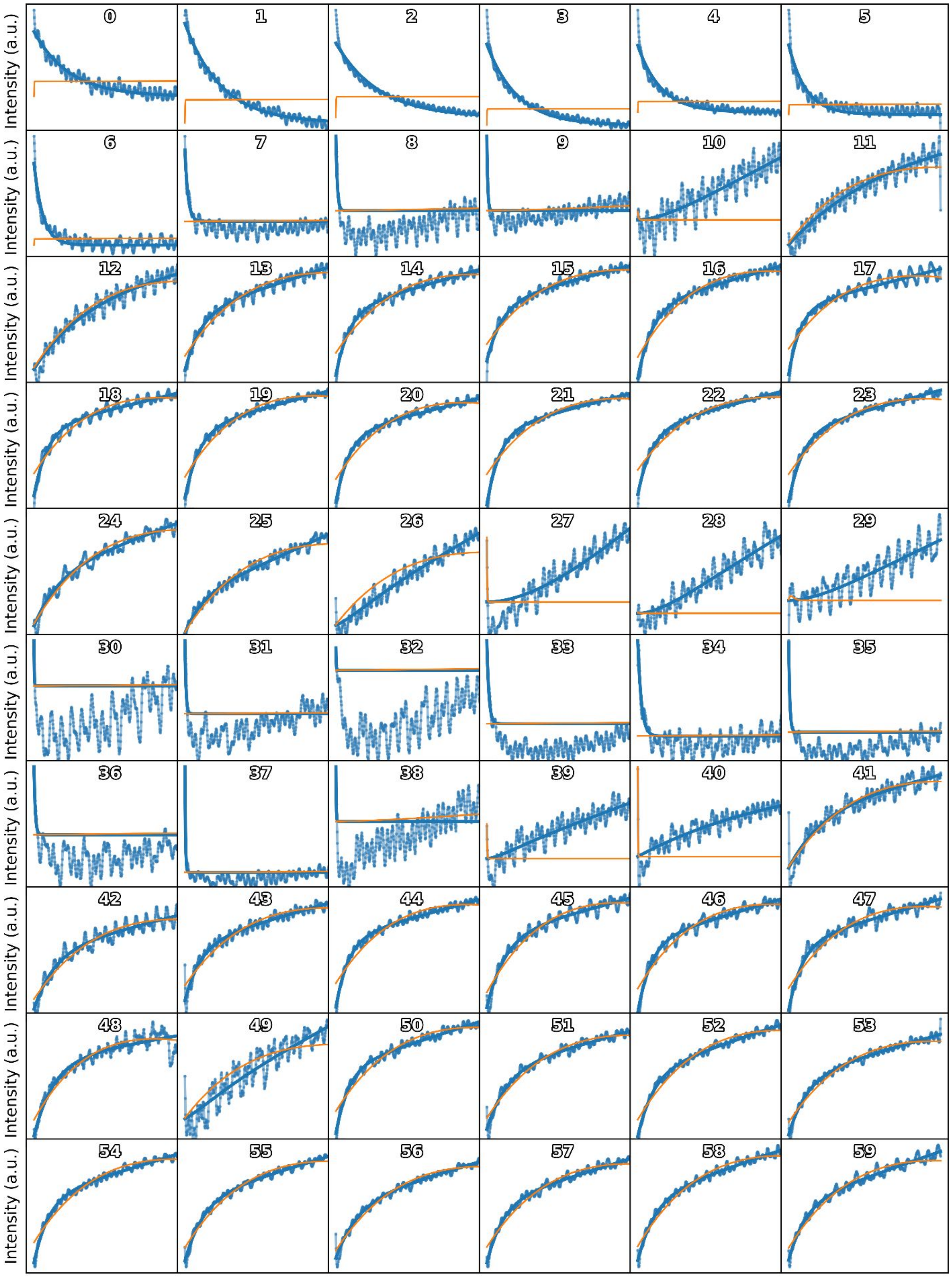
Intensity (a.u.)
0
1
2
3
4
5
6
7
8
9
10
11
12
13
14
15
16
17
18
19
20
21
22
23
24
25
26
27
28
29
30
31
32
33
34
35
36
37
38
39
40
41
42
43
44
45
46
47
48
49
50
51
52
53
54
55
56
57
58
59

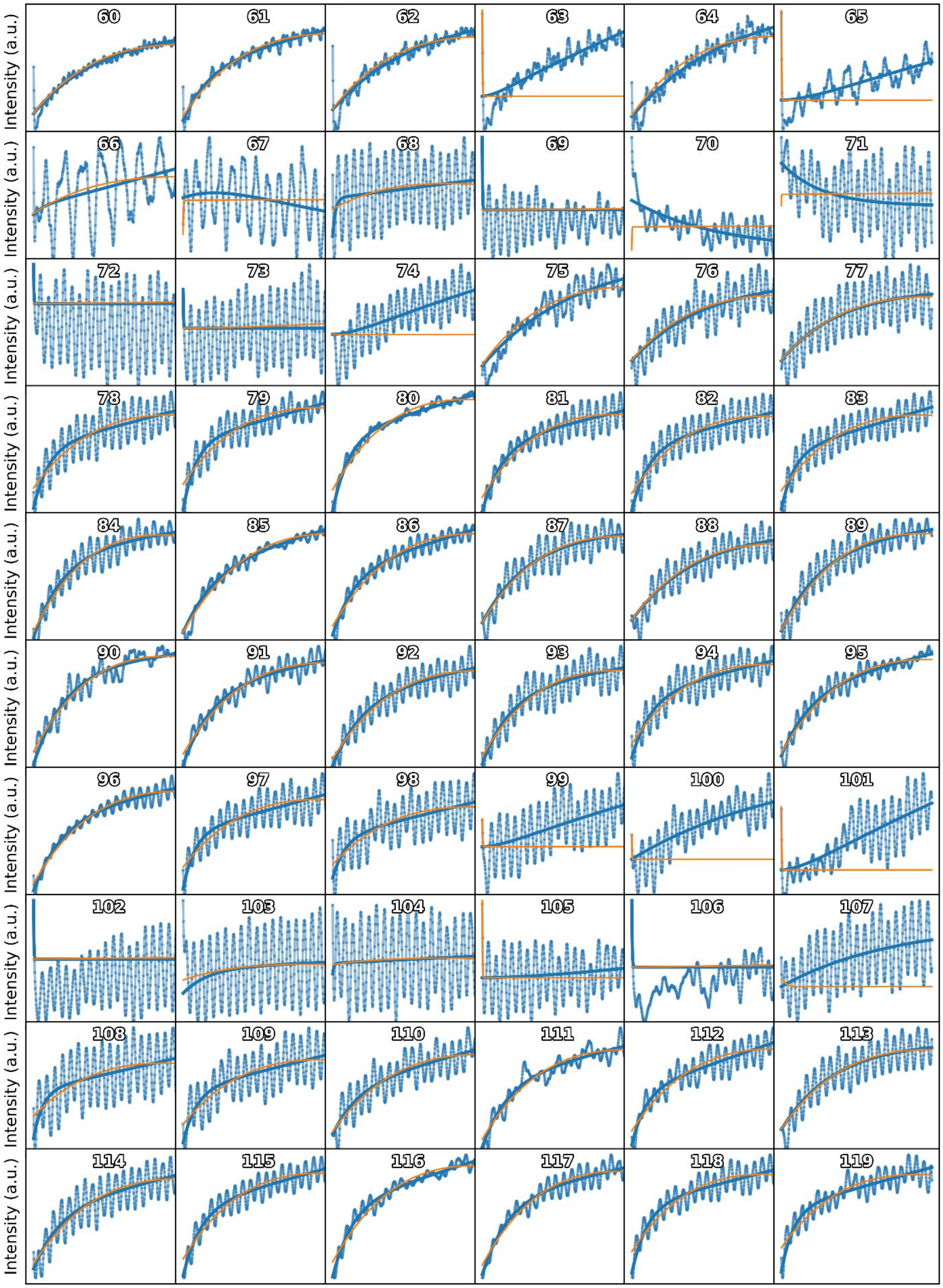
Intensity (a.u.)
60
61
62
63
64
65
66
67
68
69
70
71
72
73
74
75
76
77
78
79
80
81
82
83
84
85
86
87
88
89
90
91
92
93
94
95
96
97
98
99
100
101
102
103
104
105
106
107
108
109
110
111
112
113
114
115
116
117
118
119

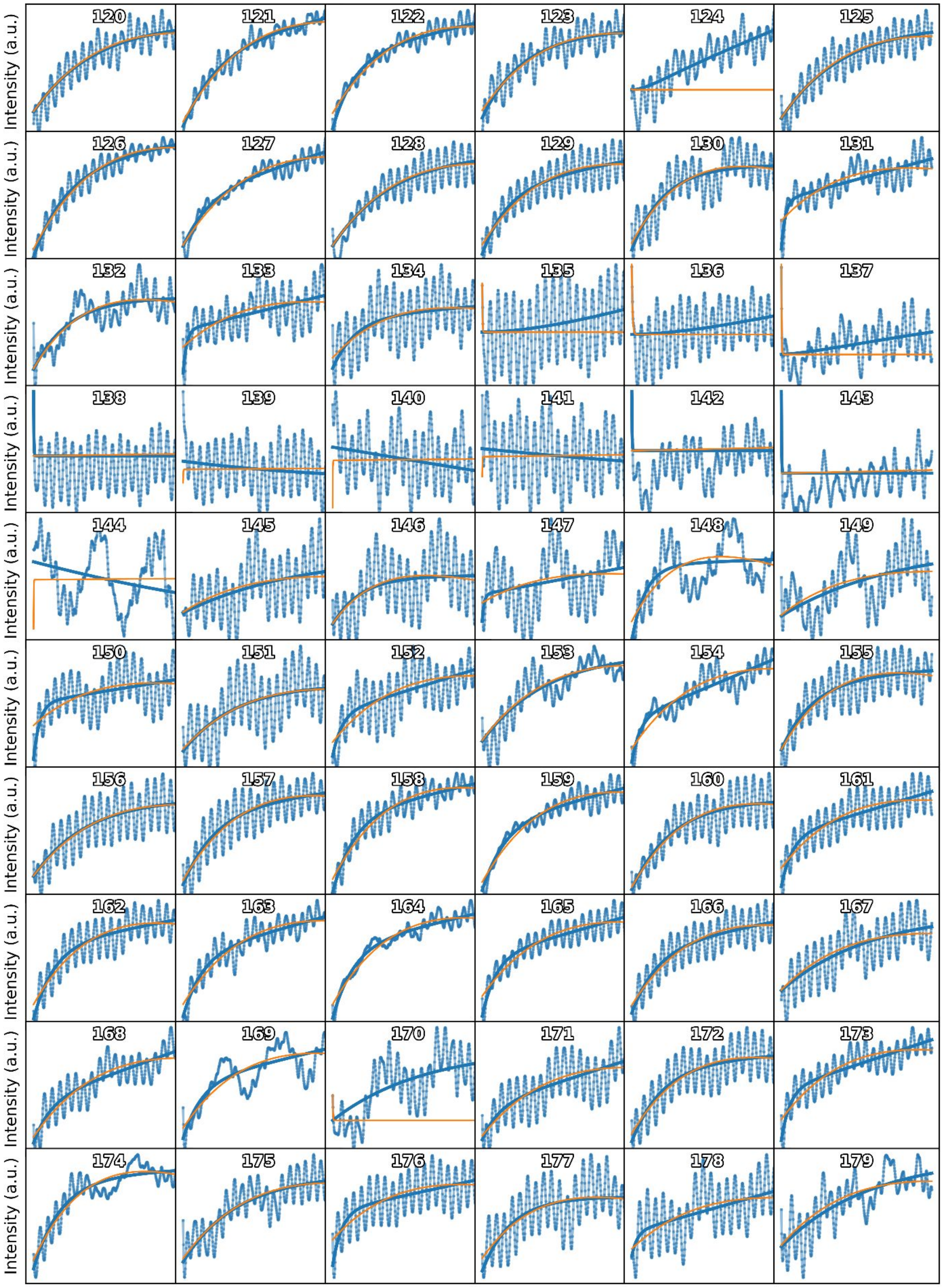

Intensity (a.u.)
120
121
122
123
124
125
126
127
128
129
130
131
132
133
134
135
136
137
138
139
140
141
142
143
144
145
146
147
148
149
150
151
152
153
154
155
156
157
158
159
160
161
162
163
164
165
166
167
168
169
170
171
172
173
174
175
176
177
178
179

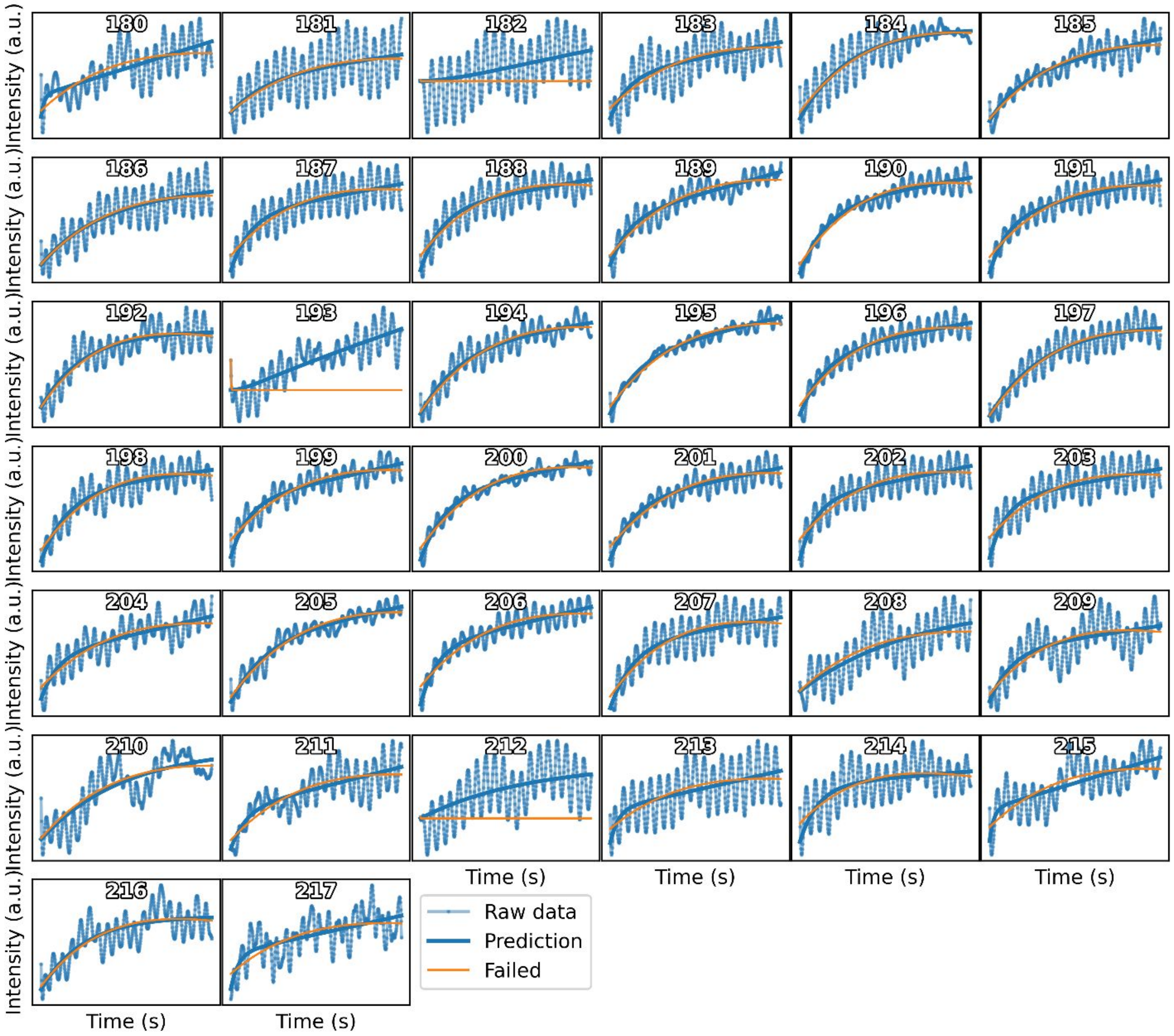

Figure S10 Fitting process with raw data, prediction curve and failed prediction curve sample treated_213nm.

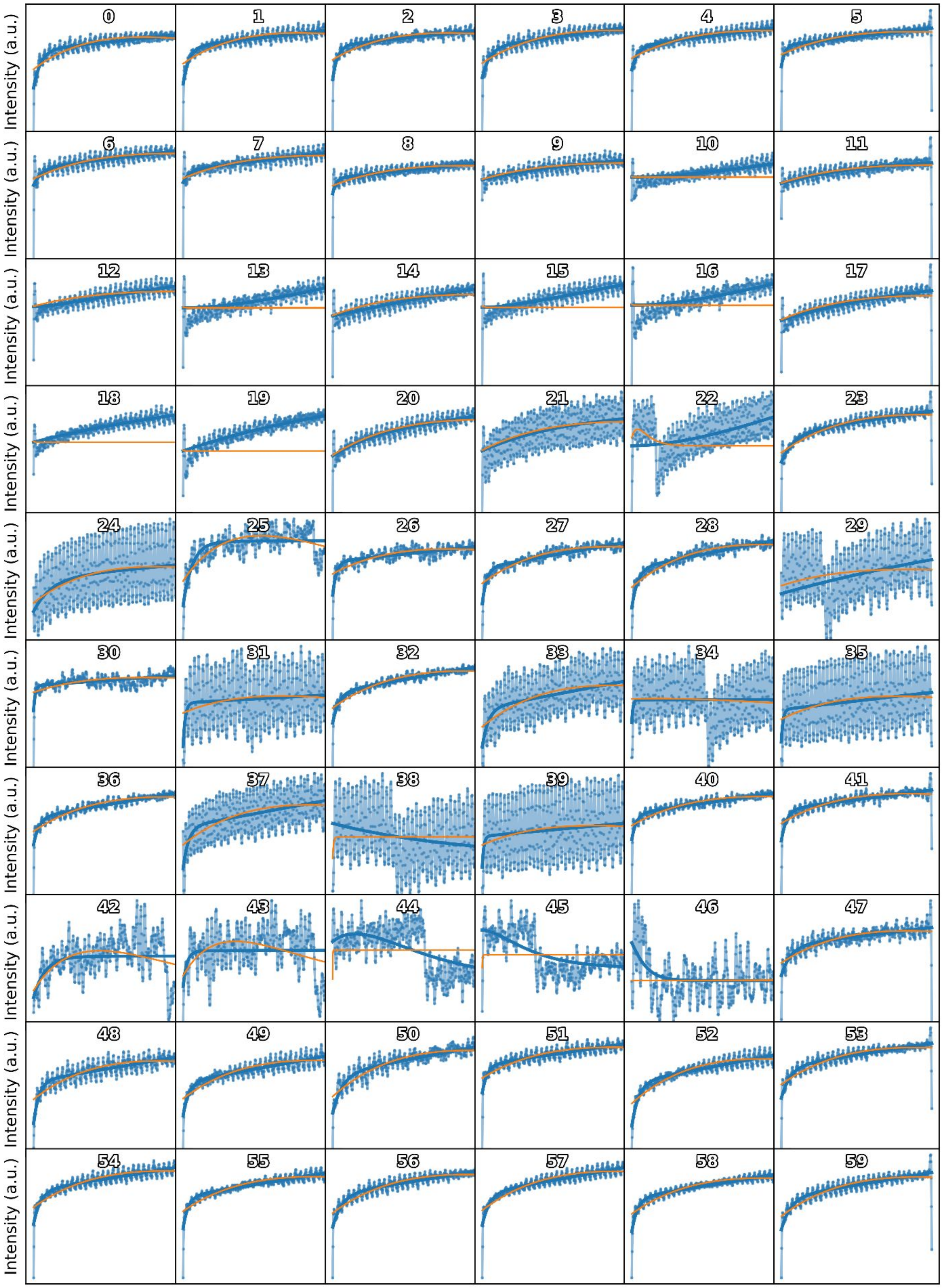
Intensity (a.u.)
0
1
2
3
4
5
6
7
8
9
10
11
12
13
14
15
16
17
18
19
20
21
22
23
24
25
26
27
28
29
30
31
32
33
34
35
36
37
38
39
40
41
42
43
44
45
46
47
48
49
50
51
52
53
54
55
56
57
58
59

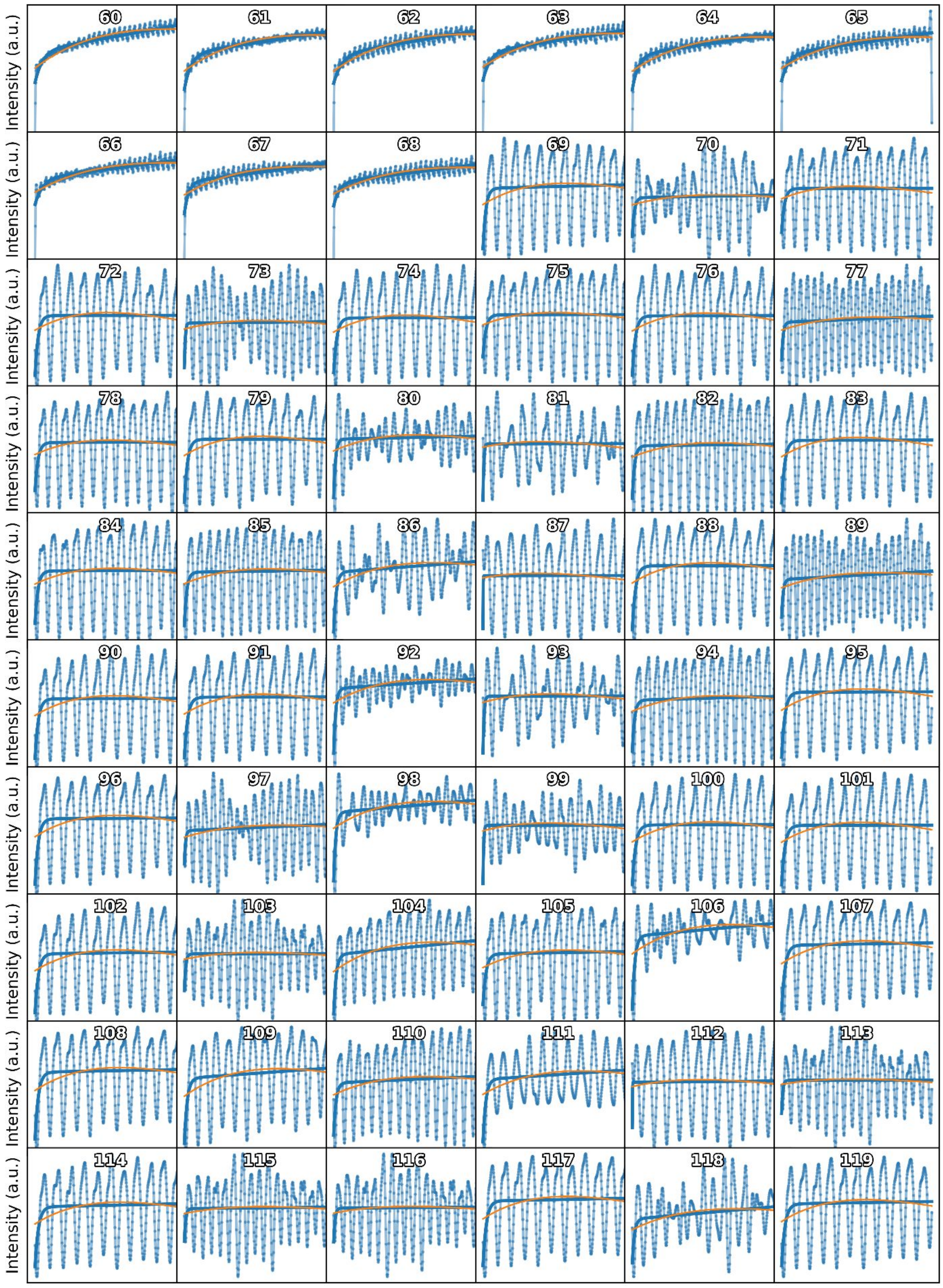
Intensity (a.u.)
60
61
62
63
64
65
66
67
68
69
70
71
72
73
74
75
76
77
78
79
80
81
82
83
84
85
86
87
88
89
90
91
92
93
94
95
96
97
98
99
100
101
102
103
104
105
106
107
108
109
110
111
112
113
114
115
116
117
118
119

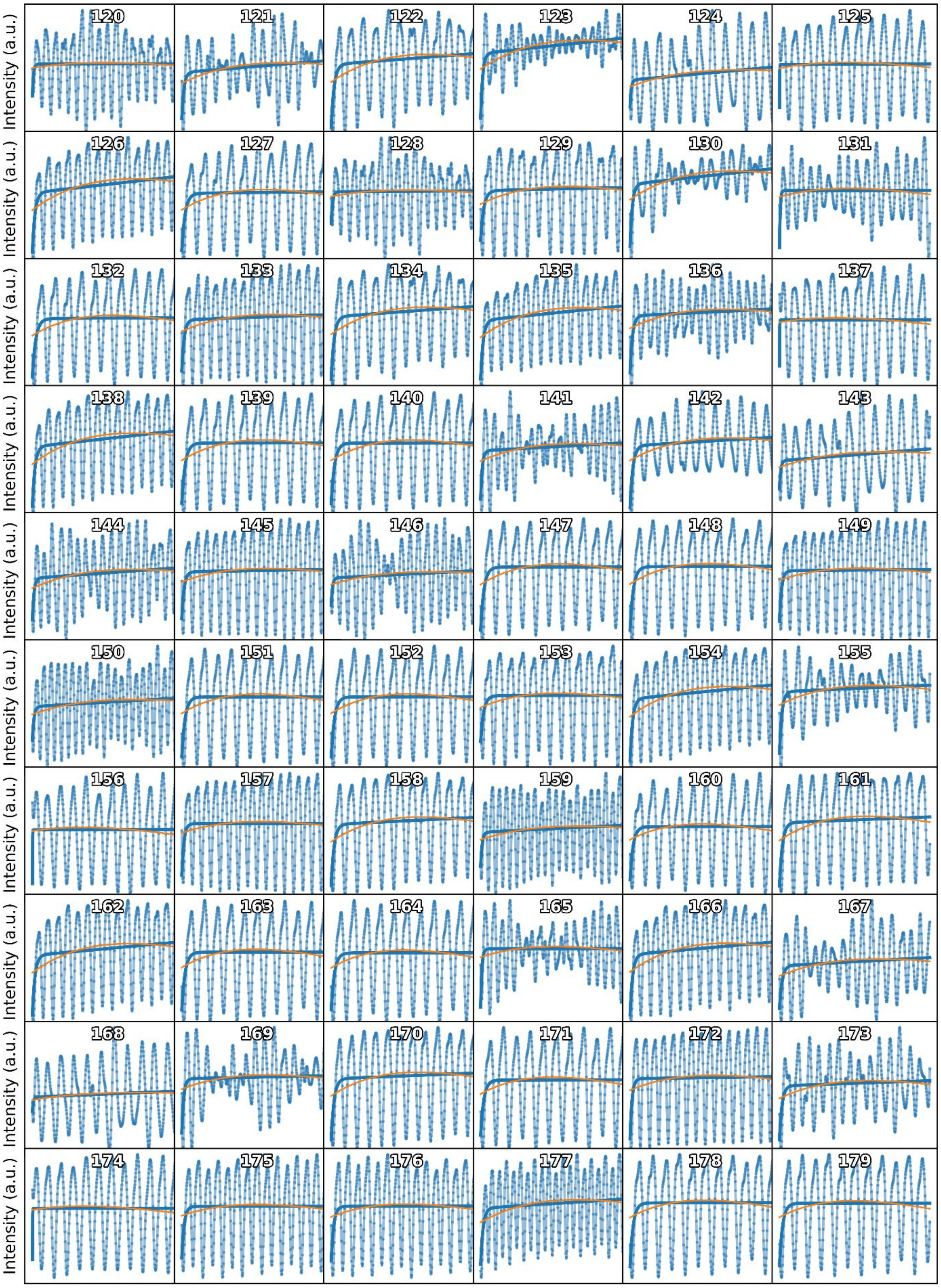

Intensity (a.u.)
120
121
122
123
124
125
126
127
128
129
130
131
132
133
134
135
136
137
138
139
140
141
142
143
144
145
146
147
148
149
150
151
152
153
154
155
156
157
158
159
160
161
162
163
164
165
166
167
168
169
170
171
172
173
174
175
176
177
178
179

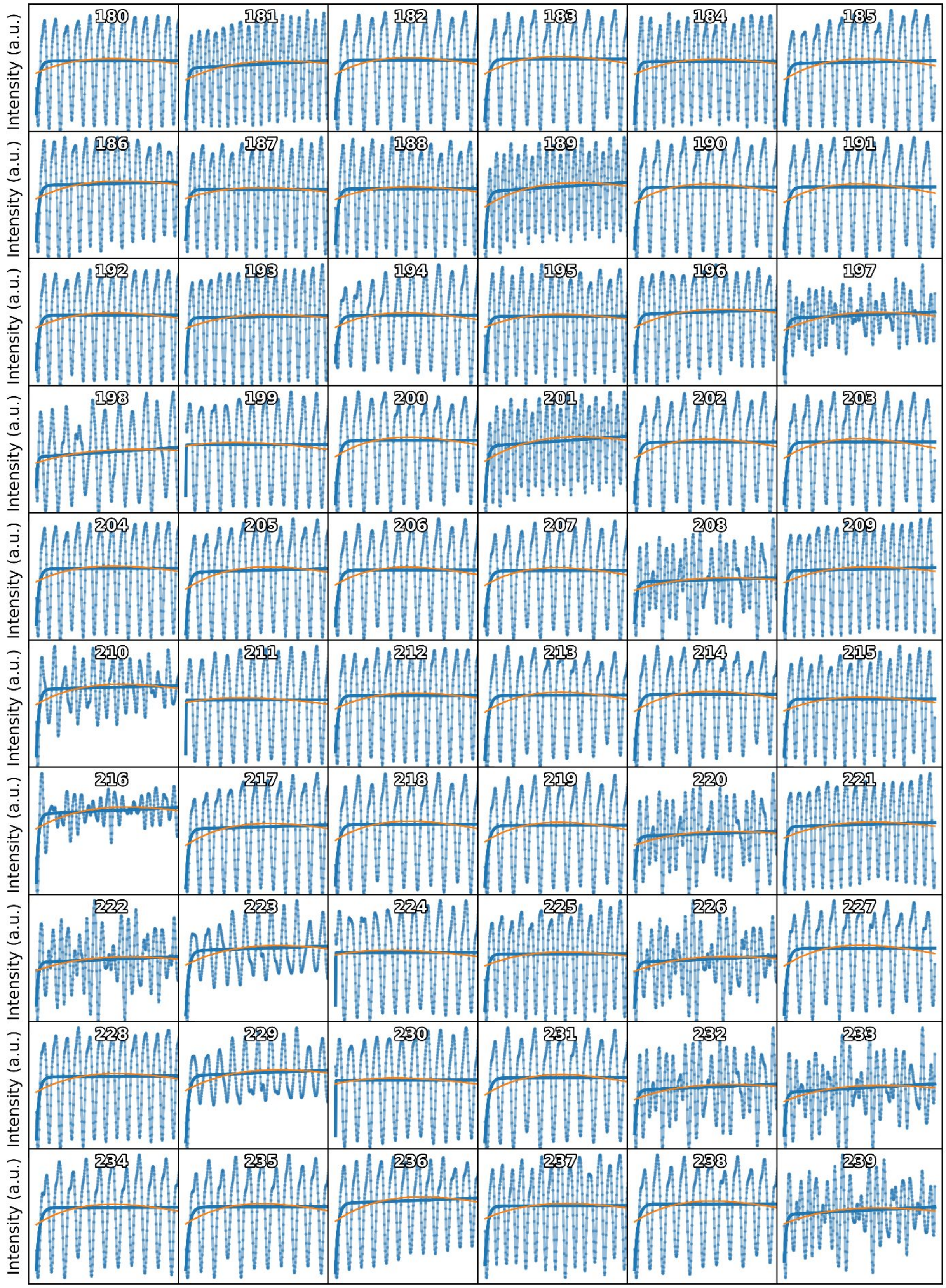

Intensity (a.u.)
180
181
182
183
184
185
186
187
188
189
190
191
192
193
194
195
196
197
198
199
200
201
202
203
204
205
206
207
208
209
210
211
212
213
214
215
216
217
218
219
220
221
222
223
224
225
226
227
228
229
230
231
232
233
234
235
236
237
238
239

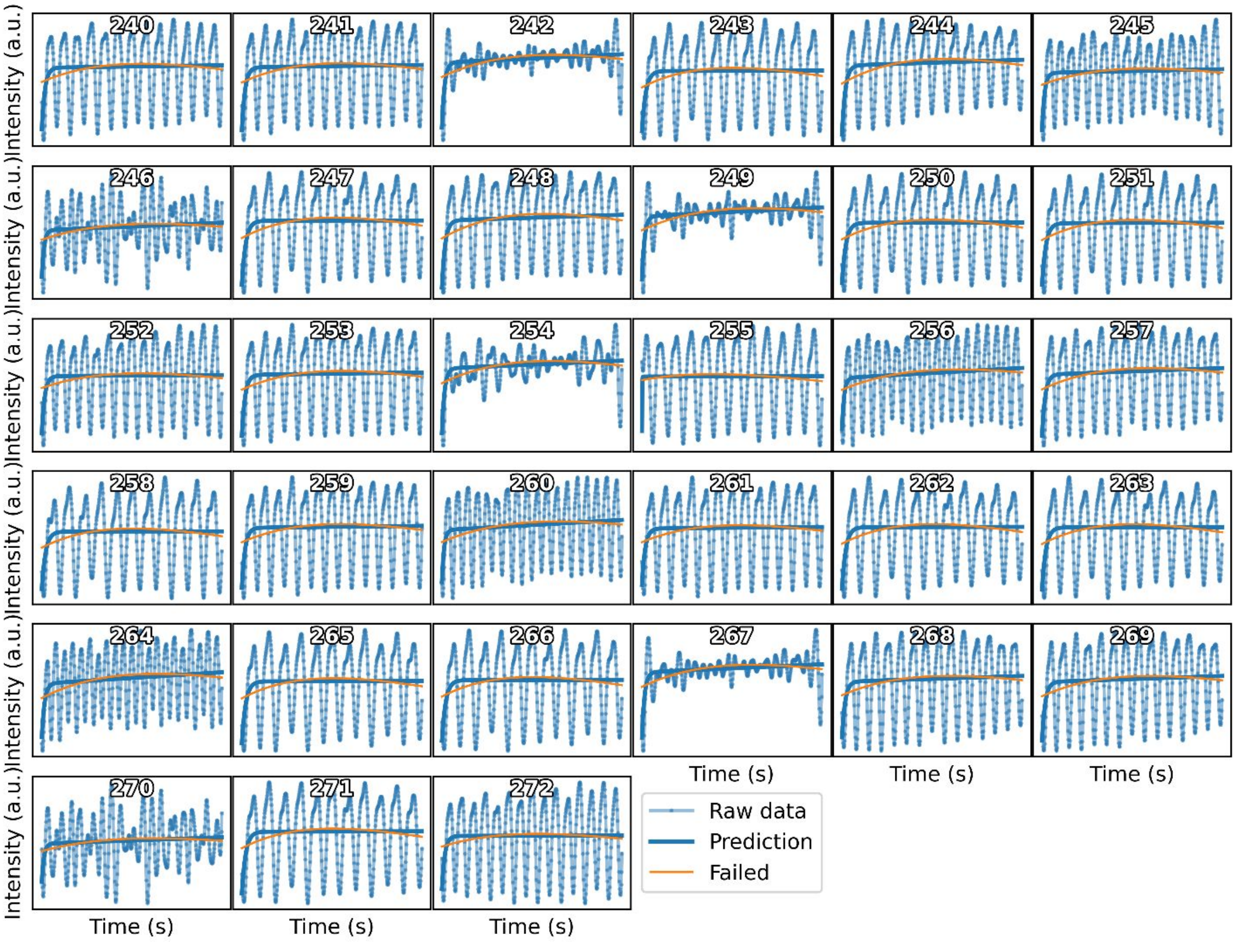

Figure S11 Fitting process with raw data, prediction curve and failed prediction curve sample treated_162nm.

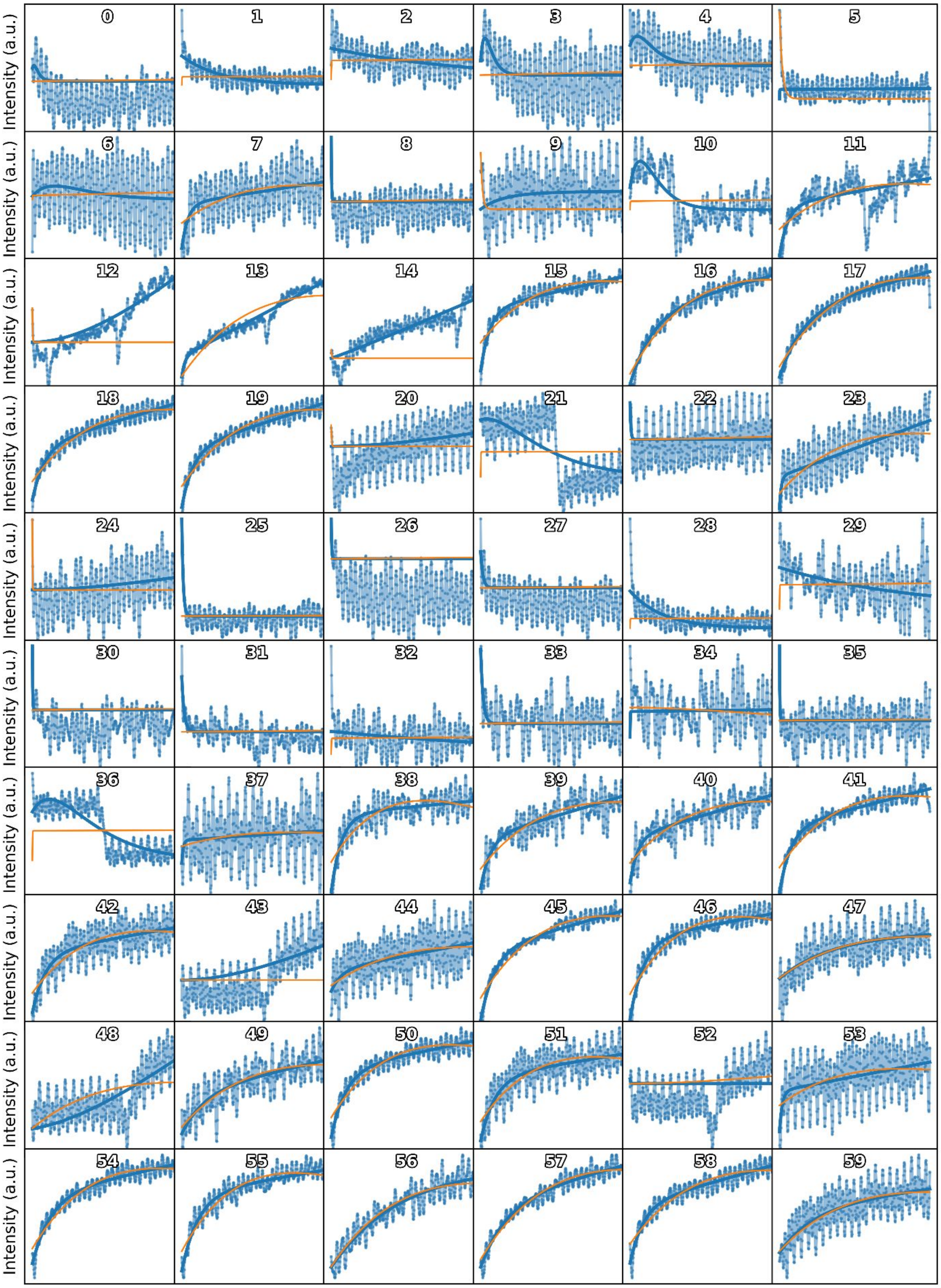

Intensity (a.u.)
0
1
2
3
4
5
6
7
8
9
10
11
12
13
14
15
16
17
18
19
20
21
22
23
24
25
26
27
28
29
30
31
32
33
34
35
36
37
38
39
40
41
42
43
44
45
46
47
48
49
50
51
52
53
54
55
56
57
58
59

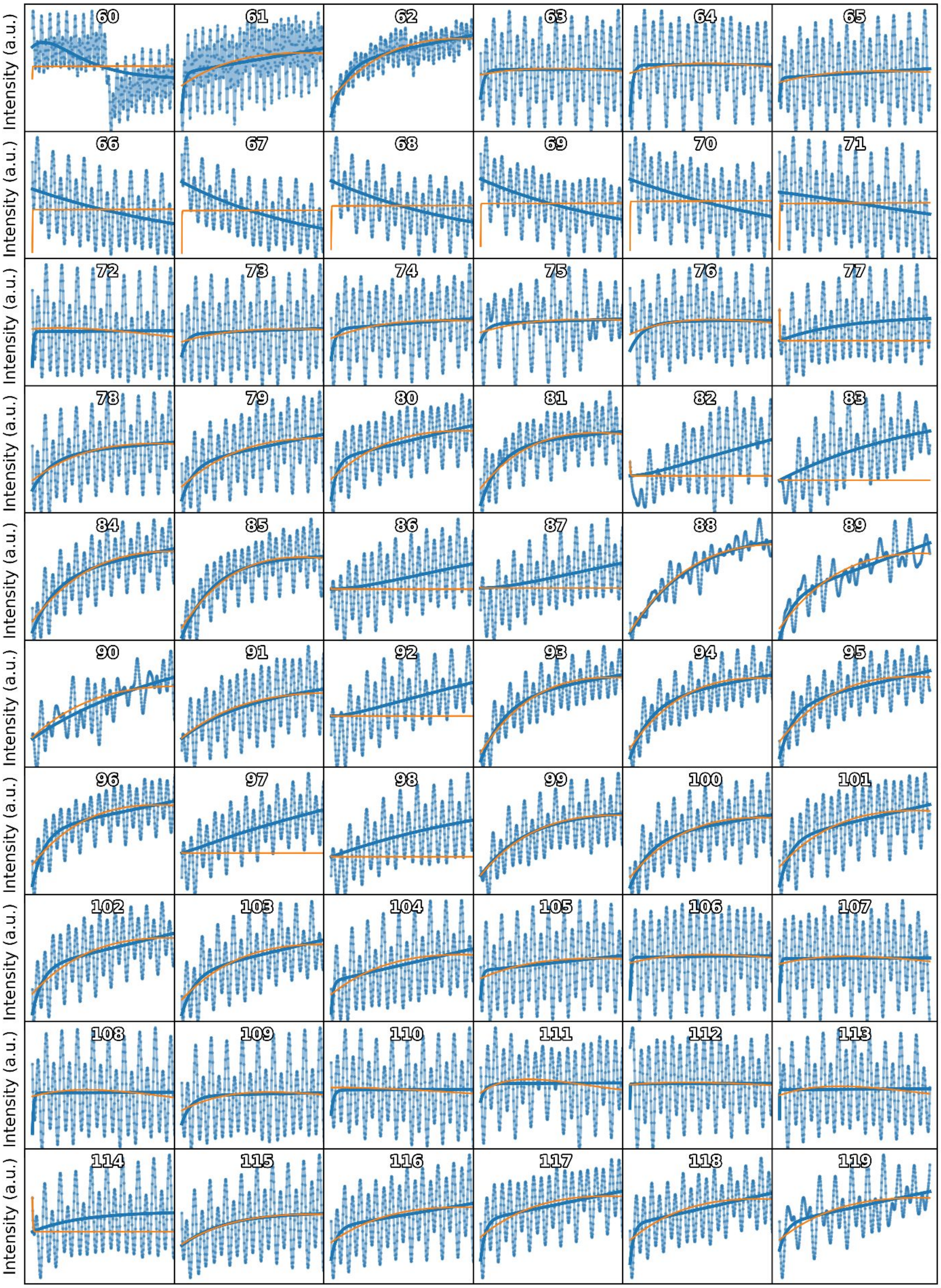
Intensity (a.u.)
60
61
62
63
64
65
66
67
68
69
70
71
72
73
74
75
76
77
78
79
80
81
82
83
84
85
86
87
88
89
90
91
92
93
94
95
96
97
98
99
100
101
102
103
104
105
106
107
108
109
110
111
112
113
114
115
116
117
118
119

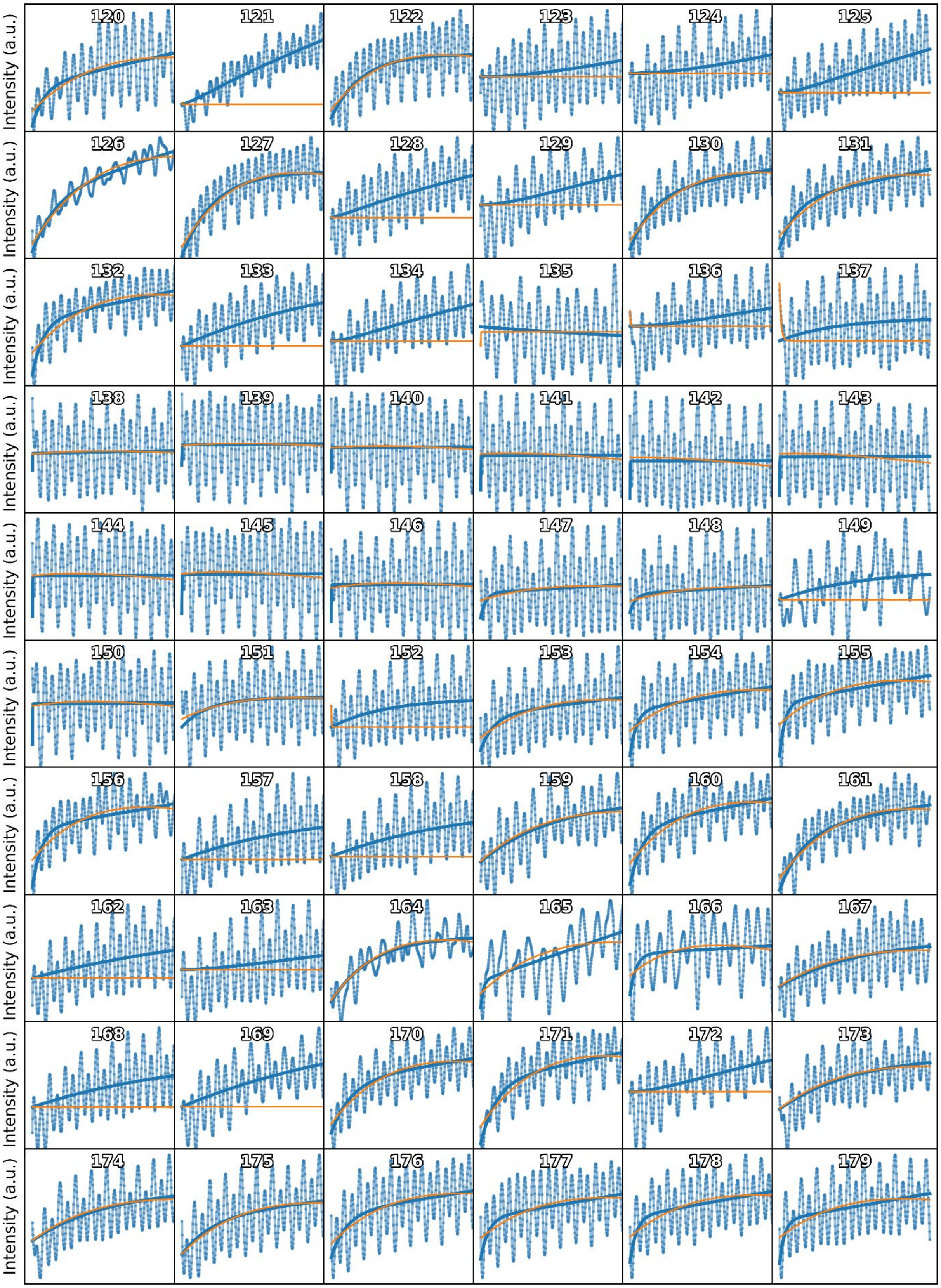
Intensity (a.u.)
120
121
122
123
124
125
126
127
128
129
130
131
132
133
134
135
136
137
138
139
140
141
142
143
144
145
146
147
148
149
150
151
152
153
154
155
156
157
158
159
160
161
162
163
164
165
166
167
168
169
170
171
172
173
174
175
176
177
178
179

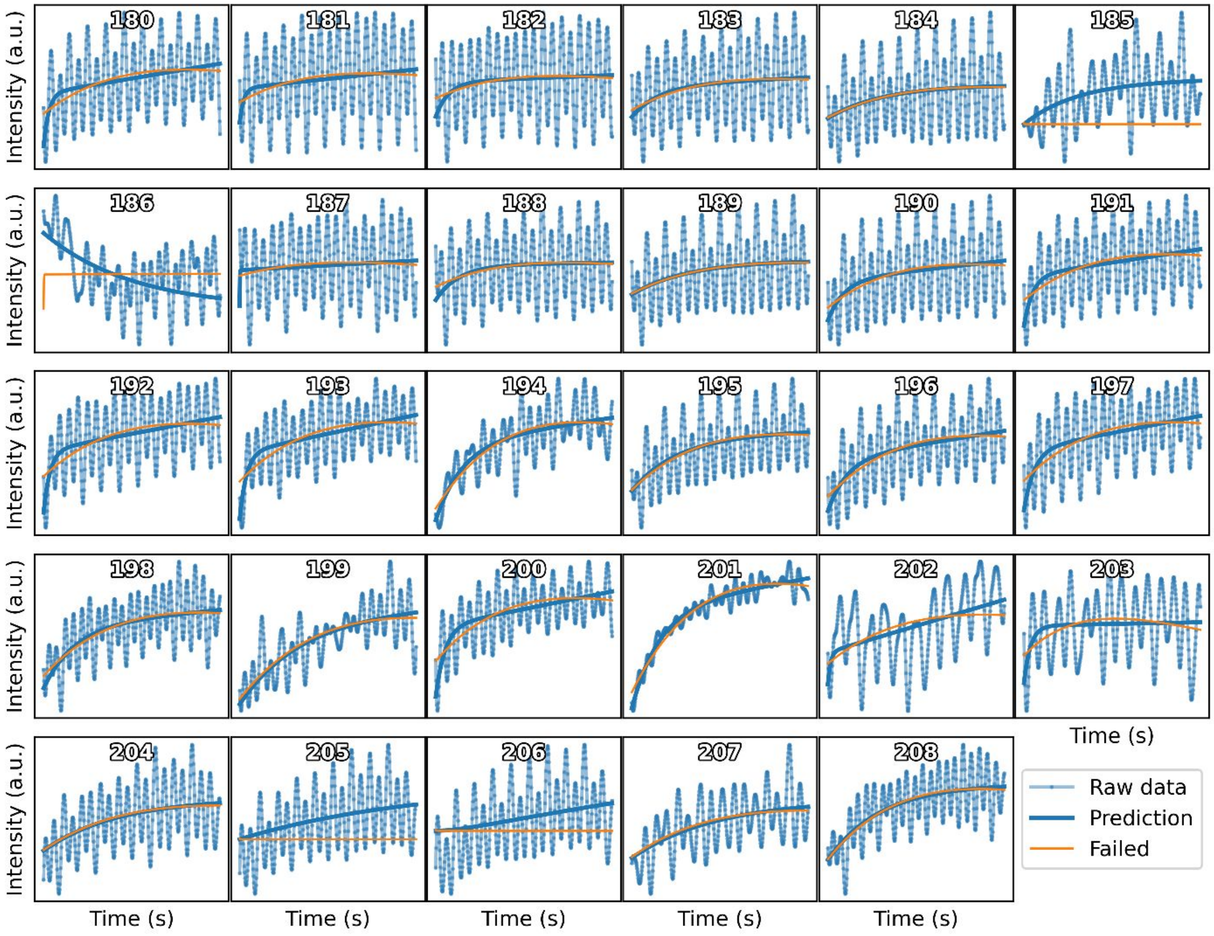

Figure S12 Fitting process with raw data, prediction curve and failed prediction curve sample untreated_81nm.
